# Pediatric emergency medicine in Lebanon: Physician-perceived comfort levels, challenges, and educational needs

**DOI:** 10.1371/journal.pone.0361212

**Published:** 2026-11-02

**Authors:** Nabil Moughnieh, Linda Abou-Abbas, Tom Beattie, Laura Smith

**Affiliations:** 1 Pediatrician/Neonatologist at Rafic Hariri University Hospital, Baabda, Lebanon; 2 Faculty of Medical Sciences, Lebanese University, Beirut, Lebanon; 3 International Committee of the Red Cross, Beirut, Lebanon; 4 INSPECT-LB (Institut National de Santé Publique Epidémiologie Clinique Et Toxicologie-Liban), Beirut, Lebanon; 5 Program Director, The University of Edinburgh, Edinburgh, United Kingdom; 6 Deputy Program Director, The University of Edinburgh, Royal Hospital for Children and Young People Edinburgh, Edinburgh, United Kingdom; Northwestern University Feinberg School of Medicine, UNITED STATES OF AMERICA

## Abstract

**Background:**

Pediatric Emergency Medicine (PEM) is a rapidly evolving subspecialty that improves the quality and safety of care for children. Despite global advancements, PEM remains underdeveloped in many low- and middle-income countries, including Lebanon. Despite a large pediatric population there is no formal paediatric emergency medicine system in place, and care largely delivered by physicians practicing without standardized or specialized training. This lack of dedicated PEM training and organized emergency care pathways may compromise the timely recognition and management of pediatric emergencies, potentially affecting patient outcomes.

**Objectives:**

The present study aimed to assess physicians perceived comfort in managing pediatric emergencies, identify training gaps, and explore systemic and institutional challenges affecting pediatric emergency care in Lebanon.

**Methods:**

A descriptive cross-sectional survey was conducted among physicians involved in pediatric emergency care across Lebanon. The questionnaire assessed self-reported comfort in managing pediatric emergencies, perceived challenges, and priorities for training and PEM workforce development.

**Results:**

Responses from 59 physicians were analysed. Most were male 38 (64.4%), with a median clinical experience of 7 years. General pediatricians represented 28 (47.5%), emergency physicians 17 (28.8%), PEM specialists 8 (13.6%), and pediatric intensivists 6 (10.2%). Physicians reported high perceived comfort in pediatric fluid management 52(88.1%), chest X-ray interpretation 46 (77.9%), and general pediatric emergencies 42 (71.2%), but lower comfort in procedural sedation, suturing 35 (59.6%), fracture stabilization 35 (59.4%), and Extended Focused Assessment Sonography in Trauma (EFAST) 22 (41.8%). General pediatricians reported greater comfort with general pediatric emergencies, while emergency-focused physicians had higher comfort with trauma-related skills and procedural interventions. Major challenges included gaps in practical and theoretical training, limited hospital infrastructure, shortages of devices and human resources, and the need for continuing medical education. Nearly all participants 57 (96.6%) supported the establishing a dedicated PEM fellowship, highlighting training priorities in trauma, medical emergencies, life support, procedural skills, and pediatric imaging.

**Conclusion:**

Pediatric emergency care in Lebanon is constrained by gaps in skills, training, and systemic resources. There is an urgent need to establish standardized PEM training pathways, enhance curricula, and strengthen system-level preparedness. Implementing these measures will support a competent PEM workforce and improve the quality and safety of emergency care for children nationwide.

## 1. Introduction

Pediatric Emergency Medicine (PEM) is a rapidly advancing subspecialty focused on the urgent assessment and management of critically ill or injured children across diverse emergency care settings [1]. Children represent approximately 25% of Emergency Department (ED) visits globally [2], and their care is frequently provided not only by pediatricians and family physicians but also by emergency doctors without specialized pediatric training [1–3]. The presence of PEM-trained consultants has been shown to significantly improve clinical outcomes, including reducing hospitalization rates by 27% [2], underscoring the importance of specialized pediatric expertise in the ED.

PEM first emerged as a distinct discipline in the early 1980s in the United States, with formal accreditation and board certification processes established during the following decade [1,4,5]. Two recognized pathways currently exist for PEM specialization—pediatrics-based and emergency medicine-based—each designed to ensure robust exposure to pediatric emergencies and critical care [6]]. However, a universally agreed-upon core PEM curriculum remains lacking for emergency medicine (EM) residents, and various studies have highlighted important gaps in pediatric exposure throughout training [7,8]. Similar structured PEM subspecialty programs have since evolved in Canada, the United Kingdom (UK), Ireland, and Australia/New Zealand through national standardization efforts and the creation of multi-center research networks such as Pediatric Emergency Research Canada (PERC) and Pediatric Emergency Research UK and Ireland (PERUKI), and the Paediatric Research in Emergency Departments International Collaborative (PREDICT) in Australia and New Zealand, established in 2004 [9–11]. Across Europe, PEM development remains inconsistent, with limited formal recognition in a number of countries [3]. In Latin America, PEM has emerged as an evolving subspecialty aimed at addressing regional pediatric emergency care needs, although considerable variability persists in curricula and training methodologies. Between 2010 and 2015, countries including Uruguay, Paraguay, Peru, Costa Rica, the Dominican Republic, and Argentina formally recognized PEM as a subspecialty, reflecting ongoing regional efforts to improve pediatric emergency care and establish more standardized training programs [12].

Training in PEM requires a curriculum that integrates clinical exposure with effective educational strategies, including simulation-based learning, hands-on procedural training, e-learning, and competency-based assessments [4,5,13–20]. Core pediatric-specific procedures such as airway management, trauma resuscitation, chest tube, procedural sedation, vascular access, and neonatal stabilization are fundamental to PEM skills development. In addition, competence in pediatric clinical reasoning [21] and effective communication with children and their families is also essential for safe and high-quality care, given children’s distinct physiology, patterns of illness presentation, and age-dependent communication needs compared with adults [22–28]. Beyond clinical skills, PEM training should also incorporate emergency department leadership and management competencies, including team coordination, patient flow, and resource utilization, which are integral to effective emergency care delivery.

Globally, inequities remain pronounced. Many low- and middle-income countries (LMICs) grapple with a shortage of trained pediatric emergency specialists, limited pediatric-specific infrastructure, and insufficient standardized training programs [8,15]. Up to 90% of pediatric deaths in LMICs are linked to deficiencies in emergency care delivery [8]. International organizations including WHO and IFEM have therefore emphasized the urgent need to strengthen PEM services and develop local training capacity tailored to resource-constrained health systems [8,15].

Lebanon provides a compelling example of PEM underdevelopment within a strained healthcare system. Political instability, economic collapse, a large refugee population, the COVID-19 pandemic, and the 2020 Beirut Port explosion have collectively weakened health infrastructure [29]. Despite nearly one-third of the population being children [30], PEM remains a nascent subspecialty within Lebanese ED. Only 27% of EDs report having attending physicians available 24/7 to manage pediatric cases [31], and as of January 2025, no physicians are formally registered as PEM specialists with the Lebanese Order of Physicians. Consequently, pediatric emergency care is largely delivered by general pediatricians or emergency physicians without structured PEM training, resulting in gaps in essential competencies such as trauma resuscitation, procedural sedation, and airway management.

The burden of pediatric disease in Lebanon also differs from high-income settings, with infectious and acute medical conditions being the most common reasons for ED visits rather than injury-related cases [14]. Existing evaluations have documented substantial disparities in pediatric emergency preparedness across institutions, including variability in staffing, access to pediatric-specific protocols, services, and appropriately sized equipment [14]. These persistent deficiencies underscore the urgency of strengthening pediatric emergency capabilities and establishing a standardized national framework for PEM education and workforce development.

Given these contextual challenges and the critical role of PEM in improving child health outcomes, it is imperative to assess the current landscape of pediatric emergency care in Lebanon. Understanding physicians’ comfort levels, identifying systemic and institutional barriers, and delineating training needs will provide an evidence base to guide the development of standardized, context-specific PEM educational programs. This endeavor aims to strengthen the PEM workforce, enhance care quality, and reduce preventable pediatric morbidity and mortality in Lebanon.

## 2. Methods

### 2.1 Study design

This quality improvement study employed a descriptive cross-sectional survey using a structured online questionnaire to assess physicians’ self-reported comfort, challenges, and educational needs related to PEM across EDs in Lebanon.

### 2.2 Study population and settings

The study population included postgraduate physicians involved in pediatric emergency care in Lebanon. Eligible participants were: [1] pediatricians working in general or pediatric emergency departments, [2] emergency medicine specialists, or [3] physicians with specific training or clinical experience in PEM. Physicians who were not routinely involved in pediatric emergency care were excluded. As PEM is not an officially accredited subspecialty in Lebanon; eligibility was based on physicians’ self-reported involvement in pediatric emergency care, including focused clinical training or practice in pediatric emergency care settings.

#### Sampling strategy and recruitment.

Because the number of clinicians providing pediatric emergency care in Lebanon is limited and no national registry of PEM providers exists, a broad national outreach was used. Convenience sampling was therefore used to recruit physicians from both pediatric and general emergency departments. The survey was disseminated through WhatsApp groups, hospital networks, professional associations, and medical societies. Participation was voluntary, and physicians self-identified as being involved in pediatric emergency care based on the eligibility criteria described above.

### 2.3 Data collection tool and procedure

The questionnaire was developed following a review of existing PEM literature [12,26], international training standards, and recognized competency frameworks (Royal College of Emergency Medicine, UK; American Academy of Pediatrics). Initial drafting focused on domains relevant to pediatric emergency clinical practice. Content validity was assessed by one researcher, one PEM practitioner and one epidemiologist, after which minor revisions were introduced to improve clarity and relevance. A pilot test was conducted with 5 physicians to ensure comprehension before final dissemination.

The final online questionnaire was administered using the University of Edinburgh’s secure JISC Online Surveys platform. Survey data was collected between January and August 2025. The format allowed respondents to complete the survey anonymously and at their convenience.

The tool contained four primary sections:

1. **Demographics** — including gender, professional specialty, level of training, and years of experience in pediatric emergency care.
2. **Perceived Comfort Levels in Pediatric Emergency Management** — Participants rated their perceived comfort in managing a range of clinical scenarios using a 4-point Likert scale (1 = extremely uncomfortable; 4 = extremely comfortable). Domains included:
  - Trauma and injury management
  - Resuscitation (cardiac and respiratory arrest)
  - Airway management and ventilation
  - Procedural sedation and pain control
  - Undifferentiated acute illness (e.g., febrile child, respiratory distress)
3. **Challenges in Delivering Pediatric Emergency Care** — Closed and open-ended questions captured perceived barriers at the institutional (e.g., lack of pediatric equipment), professional (e.g., limited exposure), and systemic (e.g., absence of national protocols) levels.
4. **Training Needs and Educational Priorities** — Participants selected areas where additional training is needed and identified preferred training formats (e.g., simulation-based learning, workshops, continuous professional development courses).

To ensure informed participation, an information sheet and consent statement were embedded at the start of the survey, and consent was implied upon proceeding to the questionnaire.

### 2.4 Outcome measures

Three categories of outcomes were evaluated to gain a comprehensive understanding of PEM perceived preparedness:

- **Clinical Comfort Levels: Quantitative** ratings measured self-reported comfort in managing life-threatening and high-acuity pediatric scenarios. Lower scores indicated discomfort and were interpreted as areas requiring educational focus.
- **Perceived Challenges to Care Delivery:** Responses revealed systemic and institutional limitations affecting pediatric emergency capacity, including resource gaps and inadequate workforce training. The frequency of each reported challenge was analyzed to determine the most prevalent barriers across ED settings.
- **Training Gaps and Priority Competencies:** Areas with low comfort scores combined with strong participant demand for further training were identified as urgent priorities for PEM workforce development. Qualitative feedback was reviewed to highlight additional skills or structural improvements not captured in closed questions.

### 2.5 Data analysis

Data were exported from the JISC Online Surveys platform and analyzed using IBM SPSS Statistics, Version 27.0. The dataset was initially examined for completeness and consistency, and all entries were deemed valid for inclusion. Descriptive statistics were used to summarize respondent characteristics and their reported comfort levels across different pediatric emergency domains. Categorical variables were presented as frequencies and percentages, and Likert-scale variables were summarized using frequencies and percentages across response categories. Subgroup findings were presented descriptively to illustrate patterns across professional groups; no inferential statistical testing was performed given the sample size and non-probability sampling approach.

## 3. Results

A total of 71 physicians responded to the invitation to participate in the study. Based on the predefined eligibility criteria, 12 participants were excluded because they were either not pediatricians (n = 3) or were not practicing in an emergency department (n = 9). The final sample consisted of 59 physicians who were actively involved in pediatric emergency care. The exact number of individuals who received the invitation—and thus the response rate—could not be determined. In addition, no official registry exists to define the total number of physicians working in pediatric emergency care in Lebanon; therefore, the representativeness of the sample cannot be assessed.

### 3.1 Demographic and professional characteristics of the surveyed physician

Nearly two-thirds of respondents were male 38 (64.4%). Years of clinical experience ranged from 1 to 30, with a median of 7 years (IQR = 5). Table 1 demonstrates the demography and backgrounds of the participants.

**Table 1:** Demographic and professional characteristics of the surveyed physicians (N = 59).

Variable	Category	n	%
**Gender**	Male	38	64.4
	Female	21	35.6
**Years of Clinical Experience**	Median (IQR)	7(5)	
	Range	1–30 years	
**Professional Status / Specialty**	General Pediatrician	28	47.5
	Emergency Physician	17	28.8
	PEM Specialist	8	13.6
	Pediatric Intensivist	6	10.2
**Exposure to Critically Ill or Injured Children**	Daily	36	61.0
	Weekly	17	28.8
	Monthly	4	6.8
	Less frequent	2	3.4
**Primary Workplace Setting**	Pediatric Emergency Department	35	59.3
	General Emergency Department	24	40.7

*N Frequency, % Percentage, PEM Pediatric Emergency Medicine*

### 3.2 Perceived comfort levels in pediatric emergency management

Physicians perceived comfort levels in managing pediatric emergency skills varied across all respondents (S1 Table) and by specialty (Supplementary Tables 2 and 3). Among all physicians (N = 59), self-reported comfort was highest for pediatric fluid management 52(88.1%), and chest X-ray interpretation 46(77.9%). Notable gaps were observed in procedural skills including EFAST, procedural sedation, suturing, fracture stabilization, and trauma X-ray interpretation. Detailed breakdowns by skill and specialty are provided in Fig 1 and S1 Table.

**Fig 1:**
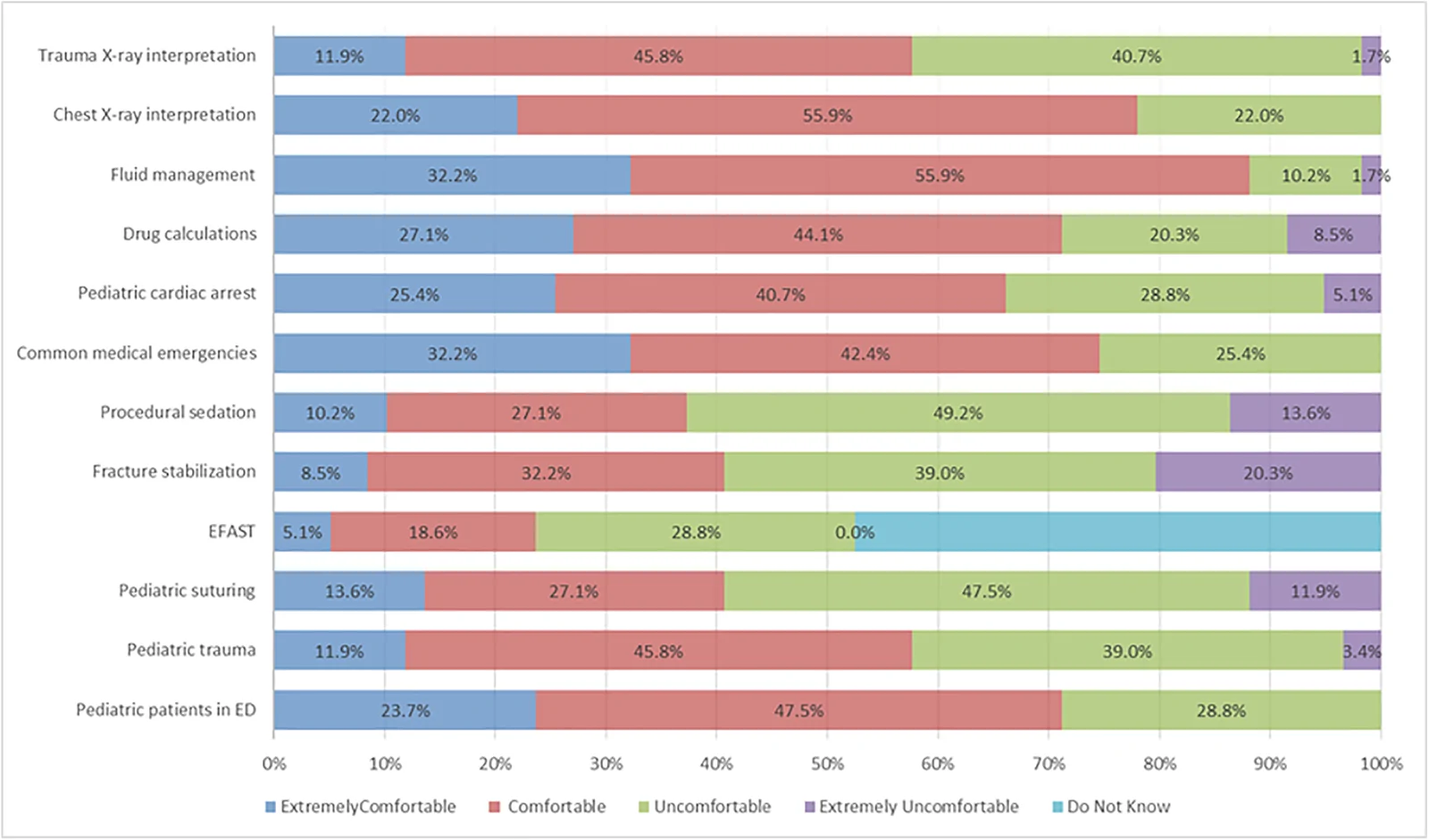
Self-reported comfort levels of respondents in managing pediatric emergencies skills (N = 59).

General Pediatricians (N = 28) reported highest comfort in managing pediatric fluid resuscitation 25(89.3%), common medical emergencies 26(92.9%), and interpreting pediatric chest X-rays 19(67.9%). Comfort was lowest for procedural sedation 6(21.4%), fracture stabilization 6(21.5%), and suturing 4(14.3%), with 17(60.7%) not knowing how to perform EFAST (S1 Table).

Emergency/Pediatric Critical Care-Focused Physicians (N = 31) demonstrated higher comfort in trauma-related skills, including trauma X-ray interpretation 25(80.7%), pediatric trauma management 24(77.4%), and fracture stabilization 18(58.1%). Lower comfort was reported for EFAST 11(35.5%) and procedural sedation 10(32.3%) (S1 Table).

General pediatricians reported higher comfort in managing pediatric patients in the emergency department (85.7% vs. 58.1%) and common pediatric emergencies (92.9% vs. 58.1%). In contrast, emergency-focused physicians reported higher comfort in pediatric trauma (77.4% vs. 35.7%), suturing (64.5% vs. 14.3%), fracture stabilization (58.1% vs. 21.4%), procedural sedation (51.6% vs. 21.4%), and trauma X-ray interpretation (80.9% vs. 32.1%) (Table 2).

**Table 2:** Descriptive comparison of perceived comfort levels in pediatric emergency skills between general pediatricians and emergency/pediatric critical care-focused physicians.

Skill	General Pediatricians (n = 28)	Emergency/pediatric critical care-Focused Physicians (n = 31)
**Pediatric patients in ED**	24(85.7%)	18(58.1%)
**Pediatric trauma**	10(35.7%)	24(77.4%)
**Pediatric suturing**	4(14.3)	20(64.5%)
**Pediatric EFAST**	5(17.9%)	9(29%)
**Fracture stabilization**	6(21.4%)	18(58.1%)
**Procedural sedation**	6(21.4%)	16(51.6%)
**Common pediatric emergencies**	26(92.9%)	18(58.1%)
**Pediatric cardiac arrest**	20/71.4%)	19(61.3%)
**Drug calculations**	23(82.1%)	19(61.3%)
**Fluid management**	25(89.3%)	27(87.1%)
**Chest X-ray interpretation**	24(85.7%)	22(71.0%)
**Trauma X-ray interpretation**	9(32.1%)	25(80.9%)

*Note: Values represent the combined percentage of participants reporting “comfortable” or “extremely comfortable” in performing each skill. n frequency, % Percentage*

### 3.3 Challenges in managing pediatric emergencies

Physicians across specialties reported multiple challenges affecting pediatric emergency care. The most frequently cited challenge was the need for additional continuing medical education (CME) and training (94.9%). Systemic barriers were also commonly reported, including lack of a functional hospital system (86.4%), human resource shortages (81.3%), limited infrastructure (79.6%), inadequate devices and supplies (76.2%), insufficient practical training (76.2%), and insufficient theoretical training (71.1%). Additionally, 67.8% of participants reported limited hospital capacity (Fig 2, S1 Table).

**Fig 2:**
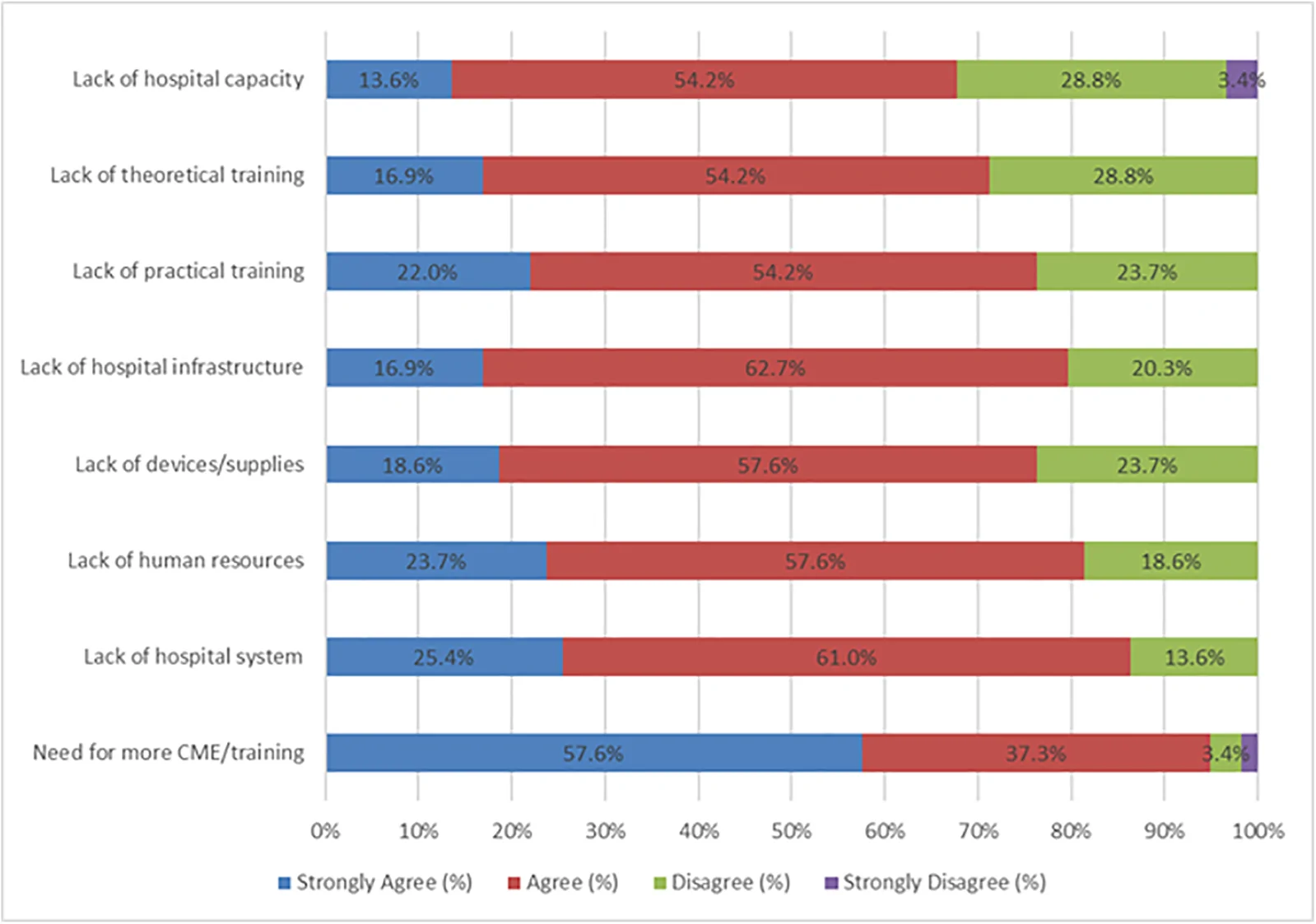
Physicians’ agreement levels on challenges in pediatric emergency care (N = 59).

When stratified by specialty, general pediatricians (N = 28) reported major training and system-level gaps, including the need for more CME and training 29(96.7%), lack of practical training 23(74.2%), deficiencies in hospital systems 24(77.4%), shortages in devices and supplies 22(71.0%), and gaps in theoretical training 22(71.0%) (S1 Table).

Emergency/pediatric critical care-focused physicians (N = 31) reported higher agreement with most challenges, particularly lack of human resources 26(92.8%), insufficient hospital infrastructure 25(89.3%), lack of devices and supplies 23(82.1%), and the need for additional CME and training 26(92.8%) (S1 Table).

Comparisons between the two groups showed similar concern levels for most challenges, with the exception of human resource shortages, reported more frequently by general pediatricians than emergency-focused physicians 26(92.9%) vs. 20(71.0%). Other differences were not statistically significant (Table 3).

**Table 3:** Descriptive comparison of perceived challenges in pediatric emergency care between general pediatricians and emergency-focused physicians (N = 59).

Challenge	General Pediatricians (n = 28)	Emergency-pediatric critical care-Focused Physicians (n = 31)
**Lack of theoretical training**	20(71.4%)	22(71.0%)
**Lack of practical training**	22(78.6%)	23(74.2%)
**Lack of hospital system**	27(96.4%)	24(77.4%)
**Lack of human resources**	26(92.9%)	22(71.0%)
**Lack of hospital infrastructure**	25(89.3%)	22(71.0%)
**Lack of hospital capacity**	20(71.4%)	20(64.5%)
**Lack of devices/supplies**	23(82.1%)	22(71.0%)
**Need for more CME/training**	26(92.9%)	30(96.8%)

n Frequency, % Percentage

### 3.4 Training needs in PEM

Nearly all respondents indicated the need for a dedicated PEM fellowship 56(96.6%). High training needs were also reported in pediatric surgical emergencies 55(94.9%), procedural sedation 54(93.2%), pediatric radiology 53(91.5%), and pediatric trauma 53(91.5%). Substantial proportions expressed the need for additional training in pediatric surgical techniques 51(88.1%), management of pediatric medical emergencies 51(88.1%), and pediatric life support 49(83.1%) (Fig 3).

**Fig 3:**
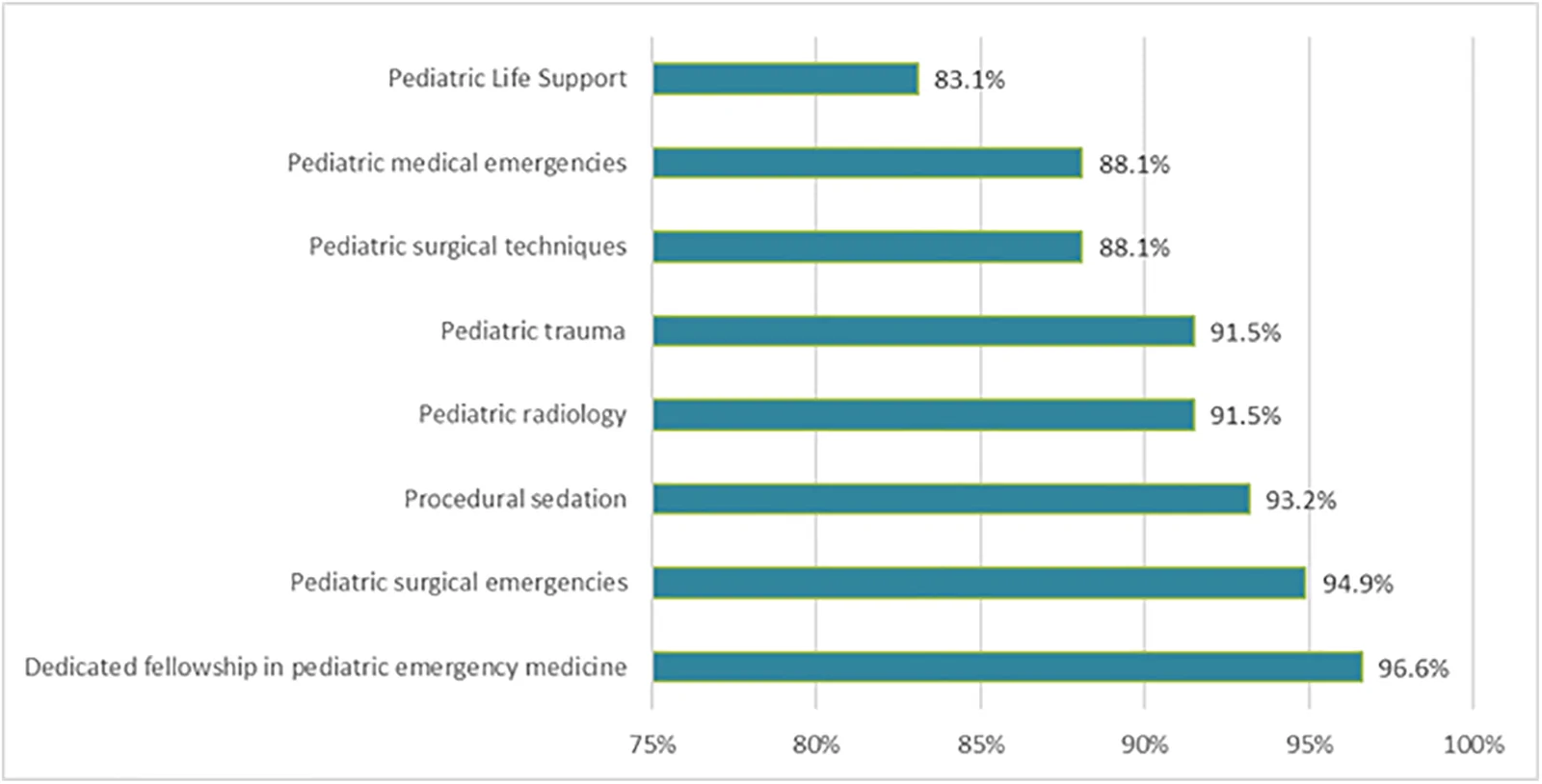
Training needs reported by physicians (N = 59).

Training needs were high across both specialties. General pediatricians (N = 28) reported greatest needs in pediatric surgical emergencies, surgical techniques, and PEM fellowship training (27, 96.4% each), followed by pediatric trauma and radiology (26, 92.9%). Emergency/pediatric critical care–focused physicians (N = 31) reported highest needs in procedural sedation and PEM fellowship training (30, 96.8% each), with similarly high needs for pediatric trauma, medical emergencies, and radiology (>90%). Across all respondents, structured PEM fellowship training, pediatric trauma and emergency management, life support, and procedural skills were identified as key priorities. All participants supported establishing a pediatric emergency fellowship in Lebanon (59, 100%) (S1 Table).

## 4. Discussion

This study assessed physicians’ self-reported comfort in managing pediatric emergencies and explored key institutional, professional, and systemic challenges, as well as training gaps and educational priorities in Lebanon. Findings reveal substantial gaps in pediatric emergency competencies, particularly in procedural skills such as sedation, suturing, fracture stabilization, and EFAST, despite high confidence in general pediatric emergency management. Specialty-specific differences were observed: general pediatricians reported greater comfort managing common pediatric emergencies, whereas emergency-focused physicians reported higher comfort in trauma care and procedural skills. Both groups reported major systemic and training challenges, including limited hospital infrastructure, shortages of devices and human resources, and insufficient theoretical and practical training. The strong support for establishing a dedicated pediatric emergency fellowship highlights the urgent need for structured training programs and capacity-building initiatives.

The variations in perceived comfort levels across pediatric emergency skills highlight important disparities in the preparedness of physicians managing acutely ill and injured children in Lebanon. The clear divide between general pediatricians and emergency-focused physicians reflects the absence of standardized pediatric emergency training pathways, resulting in uneven exposure to trauma care and procedural skills. These gaps are concerning because the procedures with lowest reported comfort —such as sedation, fracture stabilization, suturing, and point-of-care ultrasound—are essential to timely and effective emergency management. Limited competency in these areas may delay critical interventions, increase reliance on subspecialists who may not be readily available, and ultimately compromise patient outcomes. The low familiarity with EFAST among both groups is particularly notable, given that point-of-care ultrasound is now considered a core component of trauma assessment worldwide.

These findings diverge from evidence in high-income countries, where PEM is a well-established subspecialty with formalized fellowship pathways. For example, PEM fellowships in the United States have been accredited since the 1990s, leading to more consistent procedural competence and higher self-confidence among physicians staffing pediatric emergency departments [1–4]. Similar trends have been reported in Canada, Australia, and the United Kingdom, where structured PEM training has been linked to better preparedness in trauma management, ultrasound use, and procedural sedation. By contrast, studies from low- and middle-income countries report substantial gaps in pediatric resuscitation and procedural training, noting that pediatricians—particularly in peripheral or non-tertiary hospitals—often have limited exposure to critical events and lack access to simulation-based training [21,32]. This pattern aligns with our findings and suggests that the absence of national subspecialty training significantly contributes to variability in clinical readiness.

Evidence from high-income settings also demonstrates that even well-trained PEM fellows may feel underprepared for low-frequency, high-stakes procedures—such as fracture stabilization or advanced airway management—without opportunities for repeated simulation or supervised practice [23]. This underscores that skill decay occurs even in robust training environments and highlights the importance of structured reinforcement. In our study, emergency physicians—despite their specialization in acute care—reported lower comfort in managing pediatric cases compared to pediatric-trained peers, a finding consistent with previous studies showing that emergency medicine curricula often allocate limited time to pediatric rotations, resulting in insufficient exposure to pediatric pathophysiology, drug dosing, and cardiac arrest management.

Overall, these comparisons reinforce that pediatric emergency training in Lebanon remains fragmented and insufficiently standardized. The consistently low perceived comfort in trauma-related procedures and EFAST use across specialties underscores the need to integrate simulation-based learning, procedural workshops, and supervised clinical exposure into both pediatric and emergency medicine residency programs. Aligning national training pathways with international pediatric emergency competencies could help reduce skill disparities and improve the quality and safety of pediatric emergency care.

The challenges reported in this study reflect persistent systemic and educational barriers that undermine the quality of pediatric emergency care in Lebanon. The nearly collective call for more CME and structured training is consistent with evidence from resource-limited settings, where pediatricians often lack access to standardized resuscitation courses and have limited exposure to critically ill children in routine practice [32]. Similar studies from low- and middle-income countries describe parallel gaps in theoretical knowledge, procedural competency, and simulation-based training opportunities, emphasizing how insufficient preparation affects physicians’ performance during high-acuity events [8–12,14,15]. The high agreement on system-level deficiencies—including limited infrastructure, shortages of devices and supplies, and weak hospital systems—aligns with regional findings that highlight how strained health systems and resource constraints directly contribute to delays in life-saving pediatric interventions [21].

The comparable reporting of most challenges by general pediatricians and emergency-focused physicians suggests that these barriers are systemic rather than specialty-specific. The only difference—general pediatricians’ greater concern about human resource shortages—may reflect their central role in pediatric care and their greater reliance on pediatric-trained personnel, a pattern also observed in prior work on pediatric critical care capacity in similar settings [31]. These observations underscore the urgent need for coordinated efforts that strengthen hospital systems while simultaneously addressing training gaps. Aligning local practices with established pediatric emergency standards, incorporating simulation-based learning, and expanding structured PEM training programs may help mitigate the training and system-level deficits documented in this study [21,31].

The overwhelming support for establishing a dedicated PEM fellowship highlights a widely recognized gap in the current pediatric emergency training landscape in Lebanon. Physicians across specialties clearly perceive that existing residency programs do not provide the depth or breadth of skills needed to manage high-acuity pediatric emergencies, particularly in trauma care, procedural sedation, and surgical emergencies. This strong endorsement reflects a shared understanding that pediatric emergency practice requires specialized, hands-on, and simulation-based training that is not currently available through informal or on-the-job learning. Establishing a structured PEM fellowship would help standardize competencies, reduce variation in clinical practice, and strengthen national capacity to care for critically ill and injured children. It would also align Lebanon with international models of pediatric emergency training and support the development of a more skilled and confident workforce capable of providing high-quality emergency care for children. In addition, clearer delineation of responsibilities between PEM and Pediatric Intensive Care Unit (PICU) physicians would help optimize the management of critically ill children. While PEM physicians are typically responsible for the initial assessment, resuscitation, and stabilization of children in the emergency department, PICU physicians provide ongoing intensive care after admission. Defining these roles more explicitly and promoting collaborative protocols and joint training could improve coordination, reduce duplication of effort, and ensure continuity of care for critically ill pediatric patients.

### Strengths and limitations

This study addresses an important gap in the literature by providing one of the first assessments of PEM preparedness and training needs among physicians in Lebanon. In a context where published data are limited, such exploratory needs-based assessments offer valuable preliminary insights into workforce perceptions, potential skill gaps, and priority areas for educational development. The inclusion of physicians from multiple specialties involved in pediatric emergency care further broadens the perspective.

However, several limitations should be acknowledged. The use of convenience sampling and the absence of a clearly defined sampling frame limit the ability to assess representativeness. The total number of physicians invited to participate was not available; therefore, the response rate could not be determined, introducing potential non-response and selection bias. In addition, the total number of physicians working in pediatric emergency care in Lebanon is not available from official sources; therefore, the representativeness of the sample at a national level cannot be precisely determined. Because PEM is not an officially accredited subspecialty in Lebanon, eligibility relied on physicians’ self-reported involvement in pediatric emergency care, which may have introduced heterogeneity in training and experience and potential self-selection. The reliance on self-reported measures of comfort and preparedness may not accurately reflect actual clinical competence. The relatively small sample size (n = 59) further limits statistical power and generalizability. Accordingly, findings should be interpreted as reflecting the perspectives of participating physicians rather than as nationally representative estimates.

### Recommendations

To strengthen pediatric emergency care in Lebanon, a phased and pragmatic approach is needed to address both immediate educational gaps and long-term workforce development. In the short term, priority should be given to expanding CME opportunities for physicians currently practicing in pediatric emergency departments. Targeted training in point-of-care ultrasound (POCUS), interpretation of pediatric radiographs, trauma management, procedural sedation, and advanced resuscitation can help improve physicians’ confidence and the quality of care delivered in existing emergency settings. Simulation-based workshops and supervised hands-on training are particularly valuable for developing procedural competence. In parallel, pediatric and emergency medicine residency programs should incorporate structured pediatric emergency rotations and competency-based training to better prepare future clinicians. As a longer-term objective, establishing a formal PEM fellowship program would provide advanced subspecialty training and help develop a cadre of experts who can serve as clinical leaders, educators, and mentors. These fellowship-trained physicians could play a central role in sustaining CME initiatives, standardizing care, and building local training capacity. Clear delineation of responsibilities between PEM and PICU physicians should be established to ensure efficient stabilization, timely transfer, and continuity of care for critically ill children. Development of shared protocols and joint training initiatives may further strengthen coordination between these two specialties. At the system level, hospitals should improve infrastructure, ensure the availability of pediatric-specific equipment, and address workforce shortages. Finally, the development of national guidelines and competency frameworks would help standardize clinical practice and promote consistent, high-quality pediatric emergency care across institutions in Lebanon.

## Conclusion

This study highlights important gaps in pediatric emergency care as reflected by physicians’ reported comfort levels in Lebanon, particularly in trauma management, procedural skills, and point-of-care imaging. Although physicians reported higher comfort in managing common pediatric emergencies, notable disparities across specialties suggest variability in perceived comfort, likely influenced by the absence of standardized training pathways.

At the system level, shortages in human resources, limited infrastructure, and inadequate access to equipment remain major barriers to the delivery of pediatric emergency care. The strong support for establishing a dedicated PEM fellowship underscores the need for structured training opportunities tailored to the local context. Overall, this study should be interpreted as a needs assessment based on physician perceptions, providing evidence to inform future training, preparedness planning, and system strengthening efforts. Further research using objective outcome-based measures is needed to evaluate the impact of preparedness on quality of care and patient outcomes.

## Supporting information

S1 Table Supplementary tables. (DOCX)

S1 File PEM data. (XLSX)

## Abbreviations

PEM: Pediatric Emergency Medicine
ED: Emergency Department
EM: Emergency Medicine
EFAST: Extended-Focused Assessment with Sonography for Trauma
HICs: High-Income Countries
LMICs: Low- and Middle-Income Countries
WHO: World Health Organization
IFEM: International Federation of Emergency Medicine
UK: United Kingdom
PERC: Pediatric Emergency Research Canada
PERUKI: Pediatric Emergency UK and Irland
CME: Continuing Medical Education
