# Supplementary material for: Pediatric emergency medicine in Lebanon: Physician-perceived comfort levels, challenges, and educational needs
Source: PLoS One. 2026 Nov 2;21(11):e0361212. doi: 10.1371/journal.pone.0361212 (PMC13726962; doi:10.1371/journal.pone.0361212)
Supplement: Supplementary file 1 [file pone.0361212.s001.docx]

**Supplementary Tables**

**Table 1. Perceived Comfort Level of All Physicians in Pediatric Emergency Skills (N=59)**

| **Skill** | **Extremely Comfortable** | **Comfortable** | **Uncomfortable** | **Extremely Uncomfortable** | **Do Not Know** |
| --- | --- | --- | --- | --- | --- |
| **Pediatric patients in ED** | 14 (23.7) | 28 (47.5) | 17 (28.8) | 0(0) | 0(0) |
| **Pediatric trauma** | 7 (11.9) | 27 (45.8) | 23 (39.0) | 2 (3.4) | 0(0) |
| **Pediatric suturing** | 8 (13.6) | 16 (27.1) | 28 (47.5) | 7 (11.9) | 0(0) |
| **Pediatric EFAST** | 3 (5.1) | 11 (18.6) | 17 (28.8) | 0(0) | 28 (47.5) |
| **Fracture stabilization** | 5 (8.5) | 19 (32.2) | 23 (39.0) | 12 (20.3) | 0(0) |
| **Procedural sedation** | 6 (10.2) | 16 (27.1) | 29 (49.2) | 8 (13.6) | 0(0) |
| **Common pediatric emergencies** | 19 (32.2) | 25 (42.4) | 15 (25.4) | 0(0) | 0(0) |
| **Pediatric cardiac arrest** | 15 (25.4) | 24 (40.7) | 17 (28.8) | 3 (5.1) | 0(0) |
| **Drug calculations** | 16 (27.1) | 26 (44.1) | 12 (20.3) | 5 (8.5) | 0(0) |
| **Fluid management** | 19 (32.2) | 33 (55.9) | 6 (10.2) | 1 (1.7) | 0(0) |
| **Chest X-ray interpretation** | 17 (23.9%) | 41 (57.7%) | 13 (18.3%) | 0 (0.0%) | 0 (0.0%) |
| **Trauma X-ray interpretation** | 9 (12.7%) | 33 (46.5%) | 28 (39.4%) | 1 (1.4%) | 0 (0.0%) |

*Values represent frequency and percentages*

**Table 2. Perceived Comfort levels of General Pediatricians in Pediatric Emergency Skills (N=28)**

| **Pediatric Emergency Skill** | **Extremely Comfortable n (%)** | **Comfortable n (%)** | **Uncomfortable n (%)** | **Extremely Uncomfortable n (%)** | **Do Not Know n (%)** |
| --- | --- | --- | --- | --- | --- |
| **Pediatric patients in ED** | 6 (21.4%) | 18 (64.3%) | 4 (14.3%) | 0 (0%) | 0 (0%) |
| **Pediatric trauma** | 0 (0%) | 10 (35.7%) | 16 (57.1%) | 2 (7.1%) | 0 (0%) |
| **Pediatric suturing** | 0 (0%) | 4 (14.3%) | 18 (64.3%) | 6 (21.4%) | 0 (0%) |
| **EFAST** | 0 (0%) | 5 (17.9%) | 6 (21.4%) | 0 (0%) | 17 (60.7%) |
| **Fracture stabilization** | 1 (3.6%) | 5 (17.9%) | 11 (39.3%) | 11 (39.3%) | 0 (0%) |
| **Procedural sedation** | 0 (0%) | 6 (21.4%) | 17 (60.7%) | 5 (17.9%) | 0 (0%) |
| **Common medical emergencies** | 11 (39.3%) | 15 (53.6%) | 2 (7.1%) | 0 (0%) | 0 (0%) |
| **Pediatric cardiac arrest** | 7 (25.0%) | 13 (46.4%) | 6 (21.4%) | 2 (7.1%) | 0 (0%) |
| **Drug calculations** | 9 (32.1%) | 14 (50.0%) | 4 (14.3%) | 1 (3.6%) | 0 (0%) |
| **Fluid management** | 10 (35.7%) | 15 (53.6%) | 3 (10.7%) | 0 (0%) | 0 (0%) |
| **Chest X-ray interpretation** | 5 (17.9%) | 19 (67.9%) | 4 (14.3%) | 0 (0%) | 0 (0%) |
| **Trauma X-ray interpretation** | 0 (0%) | 9 (32.1%) | 18 (64.3%) | 1 (3.6%) | 0 (0%) |

*Values represent frequency and percentages*

| **Pediatric Emergency Skill** | **Extremely Comfortable**  **n (%)** | **Comfortable n (%)** | **Uncomfortable**  **n (%)** | **Extremely Uncomfortable n (%)** | **Do Not Know**  **n (%)** |
| --- | --- | --- | --- | --- | --- |
| **Pediatric patients in ED** | 8 (25.8%) | 10 (32.3%) | 13 (41.9%) | 0 (0%) | 0 (0%) |
| **Pediatric trauma** | 7 (22.6%) | 17 (54.8%) | 7 (22.6%) | 0 (0%) | 0 (0%) |
| **Pediatric suturing** | 8 (25.8%) | 12 (38.7%) | 10 (32.3%) | 1 (3.2%) | 0 (0%) |
| **EFAST** | 3 (9.7%) | 6 (19.4%) | 11 (35.5%) | 0 (0%) | 11 (35.5%) |
| **Fracture stabilization** | 4 (12.9%) | 14 (45.2%) | 12 (38.7%) | 1 (3.2%) | 0 (0%) |
| **Procedural sedation** | 6 (19.4%) | 10 (32.3%) | 12 (38.7%) | 3 (9.7%) | 0 (0%) |
| **Common medical emergencies** | 8 (25.8%) | 10 (32.3%) | 13 (41.9%) | 0 (0%) | 0 (0%) |
| **Pediatric cardiac arrest** | 8 (25.8%) | 11 (35.5%) | 11 (35.5%) | 1 (3.2%) | 0 (0%) |
| **Drug calculations** | 7 (22.6%) | 12 (38.7%) | 8 (25.8%) | 4 (12.9%) | 0 (0%) |
| **Fluid management** | 9 (29.0%) | 18 (58.1%) | 3 (9.7%) | 1 (3.2%) | 0 (0%) |
| **Chest X-ray interpretation** | 8 (25.8%) | 14 (45.2%) | 9 (29.0%) | 0 (0%) | 0 (0%) |
| **Trauma X-ray interpretation** | 7 (22.6%) | 18 (58.1%) | 6 (19.4%) | 0 (0%) | 0 (0%) |

**Table 3 Perceived Comfort levels of Emergency-Focused Physicians in Pediatric Emergency Skills (N=31)**

*Values represent frequency and percentages*

**Table 4. Challenges Reported by All Physicians (N = 59)**

| **Challenge** | **Strongly Agree n (%)** | **Agree n (%)** | **Disagree n (%)** | **Strongly Disagree n (%)** |
| --- | --- | --- | --- | --- |
| **Lack of theoretical training** | 10 (16.9%) | 32 (54.2%) | 17 (28.8%) | 0 (0.0%) |
| **Lack of practical training** | 13 (22.0%) | 32 (54.2%) | 14 (23.7%) | 0 (0.0%) |
| **Lack of hospital system** | 15 (25.4%) | 36 (61.0%) | 8 (13.6%) | 0 (0.0%) |
| **Lack of human resources** | 14 (23.7%) | 34 (57.6%) | 11 (18.6%) | 0 (0.0%) |
| **Lack of hospital infrastructure** | 10 (16.9%) | 37 (62.7%) | 12 (20.3%) | 0 (0.0%) |
| **Lack of hospital capacity** | 8 (13.6%) | 32 (54.2%) | 17 (28.8%) | 2 (3.4%) |
| **Lack of devices/supplies** | 11 (18.6%) | 34 (57.6%) | 14 (23.7%) | 0 (0.0%) |
| **Need for more CME/training** | 34 (57.6%) | 22 (37.3%) | 2 (3.4%) | 1 (1.7%) |

*Values represent frequency and percentages*

| **Challenge** | **Agree n (%)** | **Strongly Agree n (%)** | **Disagree n (%)** | **Strongly Disagree n (%)** |
| --- | --- | --- | --- | --- |
| **Lack of theoretical training** | 17 (60.7%) | 3 (10.7%) | 8 (28.6%) | 0 (0.0%) |
| **Lack of practical training** | 19 (67.9%) | 3 (10.7%) | 6 (21.4%) | 0 (0.0%) |
| **Lack of hospital system** | 21 (75.0%) | 6 (21.4%) | 1 (3.6%) | 0 (0.0%) |
| **Lack of human resources** | 23 (82.1%) | 3 (10.7%) | 2 (7.1%) | 0 (0.0%) |
| **Lack of hospital infrastructure** | 21 (75.0%) | 4 (14.3%) | 3 (10.7%) | 0 (0.0%) |
| **Lack of hospital capacity** | 17 (60.7%) | 3 (10.7%) | 7 (25.0%) | 1 (3.6%) |
| **Lack of devices/supplies** | 20 (71.4%) | 3 (10.7%) | 5 (17.9%) | 0 (0.0%) |
| **Need for more CME/training** | 13 (46.4%) | 13 (46.4%) | 1 (3.6%) | 1 (3.6%) |

**Table 5. Challenges Reported by General Pediatricians (N = 28)**

*Values represent frequency and percentages*

| **Challenge** | **Strongly Agree n (%)** | **Agree**  **n (%)** | **Disagree**  **n (%)** | **Strongly Disagree**  **n (%)** |
| --- | --- | --- | --- | --- |
| **Lack of theoretical training** | 7 (22.6%) | 15 (48.4%) | 9 (29.0%) | 0 (0.0%) |
| **Lack of practical training** | 10 (32.3%) | 13 (41.9%) | 8 (25.8%) | 0 (0.0%) |
| **Lack of hospital system** | 9 (29.0%) | 15 (48.4%) | 7 (22.6%) | 0 (0.0%) |
| **Lack of human resources** | 11 (35.5%) | 11 (35.5%) | 9 (29.0%) | 0 (0.0%) |
| **Lack of hospital infrastructure** | 6 (19.4%) | 16 (51.6%) | 9 (29.0%) | 0 (0.0%) |
| **Lack of hospital capacity** | 5 (16.1%) | 15 (48.4%) | 10 (32.3%) | 1 (3.2%) |
| **Lack of devices/supplies** | 8 (25.8%) | 14 (45.2%) | 9 (29.0%) | 0 (0.0%) |
| **Need for more CME/training** | 21 (67.7%) | 9 (29.0%) | 1 (3.2%) | 0 (0.0%) |

**Table 6. Challenges Reported by Emergency-Focused Physicians (N = 31)**

*Values represent frequency and percentages*

**Table 7. Training Needs Among General Pediatricians (N = 28)**

| **Training Area** | **Yes (%)** | **No (%)** |
| --- | --- | --- |
| **Pediatric surgical emergencies** | 96.4% | 3.6% |
| **Pediatric surgical techniques** | 96.4% | 3.6% |
| **Dedicated fellowship in PEM** | 96.4% | 3.6% |
| **Pediatric trauma** | 92.9% | 7.1% |
| **Pediatric radiology** | 92.9% | 7.1% |
| **Procedural sedation** | 89.3% | 10.7% |
| **Pediatric medical emergencies** | 85.7% | 14.3% |
| **Pediatric Life Support** | 82.1% | 17.9% |

**Table 8. Training Needs Among Emergency-Focused Physicians (N = 31)**

| **Training Area** | **Yes (%)** | **No (%)** |
| --- | --- | --- |
| **Procedural sedation** | 96.8% | 3.2% |
| **Dedicated fellowship in PEM** | 96.8% | 3.2% |
| **Pediatric surgical emergencies** | 93.5% | 6.5% |
| **Pediatric medical emergencies** | 90.3% | 9.7% |
| **Pediatric trauma** | 90.3% | 9.7% |
| **Pediatric radiology** | 90.3% | 9.7% |
| **Pediatric Life Support** | 83.9% | 16.1% |
| **Pediatric surgical techniques** | 80.6% | 19.4% |
