# Supplementary material for: Pediatric emergency medicine in Lebanon: Physician-perceived comfort levels, challenges, and educational needs
Source: PLoS One. 2026 Nov 2;21(11):e0361212. doi: 10.1371/journal.pone.0361212 (PMC13726962; doi:10.1371/journal.pone.0361212)
Supplement: Supplementary file 2 [file pone.0361212.s004.docx]

Dear Dr. Tucker,

We are pleased to resubmit the revised version of Manuscript entitled: “Pediatric Emergency Medicine Preparedness in Lebanon: Insights on Comfort, Challenges, and Educational Needs”

Ref: Submission ID: PONE-D-26-08269

We would like to thank the editor and the reviewers for their constructive and insightful comments. We have carefully addressed all comments. All responses to the reviewers’ comments are provided in italics and prefaced by “Author response.” Modifications in the manuscript are highlighted in yellow. Please find below a summary of how we addressed the comments.

My co-authors and I sincerely appreciate the opportunity to resubmit and are enthusiastic about the possibility of publication.

Nabil Moughnieh, MD

**Reviewer #1:**

This manuscript addresses an important and timely topic: pediatric emergency medicine (PEM) preparedness in Lebanon, a setting where structured PEM training pathways remain underdeveloped. The study provides valuable insights into physicians’ self-reported comfort levels, perceived challenges, and training needs, and offers a meaningful contribution to the literature on emergency care in resource-constrained settings.
Overall, the study design and analytical approach are appropriate for the research objectives. The use of a cross-sectional survey and comparison of proportions between general pediatricians and emergency-focused physicians using Chi-square or Fisher’s exact tests is suitable for the type of data collected. The findings are generally clearly presented and clinically coherent. In particular, the observed differences—where general pediatricians report higher confidence in general pediatric emergencies, and emergency-focused physicians demonstrate higher confidence in trauma-related and procedural skills—are consistent with expected differences in training and clinical exposure, supporting the internal validity of the results. That said, a few minor clarifications and improvements would strengthen the manuscript:

1- Clarification of Data Handling:

A discrepancy exists between the Data Analysis (Section 2.5) and the results presented in Tables 2 and 3. The Methods section states that ordinal variables (such as Likert-scale data) would be presented as means and standard deviations. However, the tables instead report the proportion of respondents who were “comfortable” or “extremely comfortable.” To ensure internal consistency, the Methods section should be updated to explicitly state that the Likert-scale responses were dichotomized into binary categories for analysis and presentation.

***Author’s Reply: Thank you for your valuable comment. To clarify, Likert-scale responses were presented descriptively in the tables as proportions across all response categories. For the purpose of inferential analysis, these variables were recoded into binary categories (e.g., “comfortable/extremely comfortable” versus other responses) to facilitate statistical testing. The Data Analysis section has been revised accordingly to clearly describe this approach and ensure consistency between the Methods and the results.***

2- Statistical Reporting Transparency: While the manuscript indicates that Chi-square or Fisher’s exact tests were used “as appropriate,” specifying which test was applied in each comparison (particularly where cell counts are small) would enhance transparency. It is best practice to use a footnote symbol (like * or †) on specific p-values in the tables to show exactly which test was used for each specific row.

***Author’s Reply: Thank you for this helpful suggestion. We agree that specifying the statistical test used for each comparison improves transparency. Accordingly, the tables have been revised to indicate the test applied for each p-value using appropriate footnote symbols (e.g., * for Chi-square test and † for Fisher’s exact test).***

3- Minor Typographical and Formatting Issues:
There are a few small errors in Table 2 (e.g., “0.007” and formatting inconsistencies) that should be corrected for clarity and professionalism.

***Author’s Reply: Corrected***

4- Clarity and Conciseness:
The Results section is generally well written, though minor reductions in repetition (especially where numerical values are already presented in tables) could improve readability.

***Author’s Reply: We have reviewed the Results section and reduced minor repetition by removing redundant textual descriptions of numerical values already presented in the tables.***

Overall, this is a well-conducted and relevant study that provides useful descriptive and comparative insights into pediatric emergency care capacity in Lebanon. With minor revisions to improve clarity and presentation, the manuscript would be suitable for publication.
 ***Author’s Reply:*** ***We sincerely thank the reviewer for the positive assessment of our work and for recognizing its relevance to pediatric emergency care capacity in Lebanon. We appreciate the constructive feedback provided throughout the review process, which has helped us improve the clarity and presentation of the manuscript. We have carefully addressed all comments and revised the manuscript accordingly.***

**Reviewer #2:**

This is an interesting work allowing to better understand the views of clinicians working in paediatric emergency departments in Lebanon. However I have majors comments:

- We do not know the denominator corresponding to the number of physicians working in paediatric emergency departments in Lebanon. Without this information, or an approximate one at least, it is impossible to draw any conclusions on the validity of the results. This number should be given to the reader.

***Author’s Reply: We thank the reviewer for this important comment. We agree that providing the total number of physicians working in pediatric emergency care in Lebanon would help contextualize the representativeness of our sample. However, no official or publicly available registry exists that precisely defines the number of physicians working in pediatric emergency departments nationwide, and staffing patterns vary across institutions and specialties. To address this limitation, we have clarified in the manuscript that the total national denominator is not available and have explicitly stated this as a limitation of the study. We have also expanded the discussion to emphasize that the findings should be interpreted as reflecting the perspectives of the participating physicians rather than a nationally representative sample.***

- It is surprising that the authors state that there is no PEM specialist registered in Lebanon although there is 8 in their respondents.

***Author’s Reply: The apparent discrepancy arises from differences in terminology and professional classification. In Lebanon, there is currently no formally accredited national subspecialty certification or official registry for PEM as a recognized specialty.***

***The physicians referred to as PEM in our study are clinicians who self-identified as having focused training or clinical practice in pediatric emergency or pediatric critical care settings, rather than formally registered PEM specialists in a nationally recognized subspecialty program. This has now been clarified in the manuscript to avoid ambiguity.***

- Regarding the discussion, if there is such important lacks competence among physicians actually working in emergency departments (few POCUS training, difficulties to read X-rays etc), how does the authors imagine the creation of a PEM fellowship in Lebanon? Different teaching pathways may be needed: continuing medical education at first to increase the level of care and confidence of physicians already working in the PEDs, fellowship to train the younger clinicians.

***Author’s Reply: We fully agree that the establishment of a PEM fellowship should be part of a broader, stepwise strategy rather than a stand-alone intervention. As highlighted by our findings, the presence of important gaps in procedural and diagnostic competencies among physicians currently working in pediatric emergency departments supports the need for immediate capacity-building measures. Accordingly, we have revised the Discussion to emphasize a dual approach: short-term continuing medical education (CME) programs aimed at improving current clinical competencies (e.g., POCUS, radiograph interpretation, and procedural skills), alongside the long-term development of a structured PEM fellowship to train future specialists. This integrated approach is now clearly reflected in the revised manuscript.***

It would also be interesting to know more about the work division between PEM and PICU physicians in Lebanon.

***Author’s Reply: We have addressed this point in the Discussion by highlighting the complementary roles of PEM physicians in the initial assessment, resuscitation, and stabilization of critically ill children, and PICU physicians in providing ongoing intensive care after admission. We also incorporated a recommendation emphasizing the need for clear role delineation, shared clinical protocols, and joint training initiatives to strengthen coordination and ensure continuity of care between these two specialties in Lebanon.***

Reviewer #3:

Summary
This manuscript presents a cross-sectional survey evaluating pediatric emergency medicine (PEM) preparedness, physician comfort, and training needs in Lebanon. The study addresses an important and underexplored topic, particularly in a context where structured PEM systems remain limited. The findings highlight perceived gaps in training and confidence among physicians and suggest the need for further educational and system-level development. More broadly, the study aligns with existing literature demonstrating that emergency care systems in low- and middle-income countries (LMICs) often face similar challenges, including limited subspecialty training, reliance on generalists, and variability in preparedness (Rybarczyk et al., 2020). While the manuscript provides useful preliminary insights, several methodological and interpretative issues should be addressed to strengthen its contribution.

Major Comments

1. Global Context in the Introduction

The introduction appropriately describes the development of PEM in North America and Europe. However, it would benefit from a more comprehensive global perspective. Specifically, the authors are encouraged to include the expansion of PEM training programs in Latin America and Australia, where structured educational initiatives and fellowships have developed over the past decade. Including these regions would provide a more balanced global overview and better situate Lebanon within the broader international evolution of PEM.

**Author’s Reply: Thank you for this insightful suggestion. We have revised the introduction to include the development of Pediatric Emergency Medicine (PEM) in Latin America and Australia/New Zealand. Specifically, we added information regarding the emergence of structured PEM training programs, fellowships, and collaborative research initiatives such as the Pediatric Research in Emergency Departments International Collaborative (PREDICT), as well as the expansion and recognition of PEM programs across several Latin American countries. These additions provide a more comprehensive global perspective on the evolution of PEM.**

2. Sampling Strategy and Representativeness

The study uses convenience sampling, and the total number of physicians invited to participate is not reported. As a result:
• The response rate cannot be determined
• The sampling frame is unclear
• Representativeness cannot be assessed
The statement that the sample represents “a substantial portion of the reachable pediatric emergency workforce in Lebanon” is not sufficiently supported by the data presented.
The authors should:
• Provide estimates of the national workforce (if available)
• Either justify or rephrase claims of representativeness

***Author’s Reply: We have revised the manuscript to remove the statement suggesting that the sample represents a “substantial portion” of the pediatric emergency workforce. We have clarified in the Methods section that the total number of physicians invited is unknown and that no official registry exists to define the national pediatric emergency workforce. These limitations are now explicitly acknowledged, and the findings are presented reflecting the perspectives of participating physicians rather than as nationally representative estimates.***

3. Reliance on Self-Reported Measures
The study relies on self-reported comfort levels, which may not accurately reflect clinical competence.
• Prior research shows discrepancies between confidence and actual performance (Hoysted et al., 2018)
• Self-report introduces potential reporting and recall bias
The authors should:
• Emphasize this limitation more explicitly
• Avoid interpreting “comfort” as equivalent to preparedness or competence

**Author’s Reply: *Thank you for this important comment. We agree that self-reported comfort does not necessarily reflect actual clinical competence. Accordingly, we have revised the manuscript throughout to clarify that the study assesses perceived comfort only, and we have avoided interpreting comfort as equivalent to preparedness or competence. We have also strengthened the limitations section to explicitly acknowledge the potential for reporting and recall bias associated with self-reported measures.***

4. Lack of Outcome-Based Measures The manuscript does not include objective clinical outcomes or performance indicators.

• This limits the ability to link perceived preparedness to quality of care or patient outcomes
• Similar studies emphasize the importance of outcome-based validation (Rybarczyk et al., 2020)

Recommendation:
Reframe conclusions to reflect that this is a perception-based needs assessment, not an evaluation of care quality.

***Author’s Reply:*** ***We have revised the Conclusion to clearly reflect that this study represents a perception-based needs assessment rather than an evaluation of clinical performance or care quality. We have also made statements that could imply outcome-level impact and emphasized that findings are based on reported comfort levels. In addition, we have highlighted the need for future research incorporating objective outcome measures and performance-based assessments.***

Strengths
Despite its limitations, this study has several important strengths:
• It addresses a significant gap in the literature, particularly in Lebanon
• Provides rare, context-specific data on PEM workforce perceptions
• Includes physicians from multiple relevant specialties
• Functions as a needs assessment, which is a recognized first step in health system development
Importantly, in settings with limited existing data, exploratory studies like this can play a meaningful role in:
• Identifying system gaps
• Informing future research
• Supporting academic and institutional dialogue
Such contributions are widely recognized as essential for the development of emergency care systems in LMICs (Reynolds et al., 2013; Razzak et al., 2019).
Suggested Revision – Strengths and Limitations Section
The authors may consider revising this section as follows:
This study addresses an important gap in the literature by providing one of the first assessments of pediatric emergency medicine (PEM) preparedness and training needs among physicians in Lebanon. In a context where published data are limited, such exploratory, needs-based assessments offer valuable preliminary insights into workforce perceptions, potential skill gaps, and priority areas for educational development. The inclusion of physicians from multiple specialties involved in pediatric emergency care further broadens the perspective.
However, several limitations should be acknowledged. The use of convenience sampling and the absence of a clearly defined sampling frame limit the ability to assess representativeness. The total number of physicians invited to participate is not reported, and therefore the response rate cannot be determined, introducing potential non-response bias.
In addition, the reliance on self-reported measures of comfort and preparedness may not accurately reflect actual clinical competence. The relatively small sample size (n = 59) further limits statistical power and generalizability.
These findings should therefore be interpreted as exploratory and hypothesis-generating. Future studies using larger, systematically sampled populations and objective assessments would strengthen the evidence base.

***Author’s Reply: We thank the reviewer for this thoughtful and constructive suggestion. We appreciate the revised framing of the Strengths and Limitations section, which improves clarity and better reflects the exploratory nature of the study. We have revised the manuscript accordingly, incorporating the suggested structure and wording with minor adaptations to maintain consistency with the overall manuscript style.***
