# Supplementary material for: Pediatric emergency medicine in Lebanon: Physician-perceived comfort levels, challenges, and educational needs
Source: PLoS One. 2026 Nov 2;21(11):e0361212. doi: 10.1371/journal.pone.0361212 (PMC13726962; doi:10.1371/journal.pone.0361212)
Supplement: Supplementary file 3 [file pone.0361212.s005.docx]

**Response to the Editor and Reviewer #4**

**Dear Editor,**

We sincerely thank you and Reviewer #4 for the thoughtful and constructive comments on our manuscript. The reviewer’s comments were particularly helpful in strengthening the methodological transparency of the study and ensuring that the interpretation of the findings is appropriately aligned with the study design and sample size.

In response to the comments, we have made the following major revisions to the manuscript:

**Sampling and response denominator:** We clarified that, because the survey was disseminated through multiple overlapping channels, the number of unique physicians who received the invitation could not be determined. We therefore did not calculate a response rate and have strengthened the discussion of the limitations related to sampling representativeness and potential selection bias.

**Descriptive analysis:** In accordance with the reviewer’s recommendation, we have revised the study to be presented as a descriptive cross-sectional survey. The inferential analyses and associated p-values in Tables 2 and 3 have been removed. These tables now present the findings descriptively using frequencies and percentages. The Methods, Results, and Discussion have also been revised accordingly to ensure that no statistical significance is implied by the observed subgroup differences.

**Definition of PEM and participant self-selection:** We clarified the eligibility criteria and recruitment approach in the Methods. Given the absence of a nationally recognized PEM certification or registry in Lebanon, participation relied on physicians’ self-identification as being involved in pediatric emergency care. We have explicitly acknowledged the potential for self-selection as a limitation and have avoided interpreting the sample as representative of all physicians providing pediatric emergency care in Lebanon.

**Years of clinical experience:** We clarified that years of clinical experience were collected in the survey and are reported in Table 1. The median duration of clinical experience among the 59 respondents was 7 years (IQR: 5; range: 1–30 years). Given the revised descriptive approach and limited sample size, we did not perform additional correlation or inferential analyses.

We also appreciate the reviewer’s positive observation that, even as a descriptive study, the work highlights an important area of need in pediatric emergency care. We have strengthened the manuscript to emphasize the educational and health-system needs identified by the participating physicians while maintaining a cautious interpretation and avoiding generalization beyond the study sample.

We are grateful for the opportunity to revise the manuscript and for the valuable guidance provided by the reviewer and the editorial team. We believe that the revisions have improved the methodological clarity, transparency, and overall interpretation of the manuscript, and we hope that the revised version will now be suitable for publication.

Thank you again for your time and consideration of our work.

Sincerely,

Nabil Moghnieh
On behalf of all authors
