# Supplementary material for: Pediatric emergency medicine in Lebanon: Physician-perceived comfort levels, challenges, and educational needs
Source: PLoS One. 2026 Nov 2;21(11):e0361212. doi: 10.1371/journal.pone.0361212 (PMC13726962; doi:10.1371/journal.pone.0361212)
Supplement: Peer review history

# Peer review: Decision Letter 0

**DOI:** 10.1371/journal.pone.0361212.r001

**Authors:** Jennifer Tucker

20 Apr 2026

Dear Dr. Moughnieh,

Thank you for submitting your manuscript to PLOS ONE. After careful consideration, we feel that it has merit but does not fully meet PLOS ONE’s publication criteria as it currently stands. Therefore, we invite you to submit a revised version of the manuscript that addresses the points raised during the review process.

The manuscript has been evaluated by three reviewers, and their comments are available below.

The reviewers have raised a number of concerns that need attention. They request additional information on methodological aspects of the study and clarification of some aspects of reporting.

Please submit your revised manuscript by Jun 04 2026 11:59PM. If you will need more time than this to complete your revisions, please reply to this message or contact the journal office at plosone@plos.org. When you're ready to submit your revision, log on to https://www.editorialmanager.com/pone/ and select the 'Submissions Needing Revision' folder to locate your manuscript file.

- A letter that responds to each point raised by the academic editor and reviewer(s). You should upload this letter as a separate file labeled 'Response to Reviewers'.
- A marked-up copy of your manuscript that highlights changes made to the original version. You should upload this as a separate file labeled 'Revised Manuscript with Track Changes'.
- An unmarked version of your revised paper without tracked changes. You should upload this as a separate file labeled 'Manuscript'.

If you would like to make changes to your financial disclosure, please include your updated statement in your cover letter. Guidelines for resubmitting your figure files are available below the reviewer comments at the end of this letter.

If applicable, we recommend that you deposit your laboratory protocols in protocols.io to enhance the reproducibility of your results. Protocols.io assigns your protocol its own identifier (DOI) so that it can be cited independently in the future. For instructions see: https://journals.plos.org/plosone/s/submission-guidelines#loc-laboratory-protocols. Additionally, PLOS ONE offers an option for publishing peer-reviewed Lab Protocol articles, which describe protocols hosted on protocols.io. Read more information on sharing protocols at https://plos.org/protocols?utm_medium=editorial-email&utm_source=authorletters&utm_campaign=protocols.

As the corresponding author, your ORCID iD is verified in the submission system and will appear in the published article. PLOS supports the use of ORCID, and we encourage all coauthors to register for an ORCID iD and use it as well. Please encourage your coauthors to verify their ORCID iD within the submission system before final acceptance, as unverified ORCID iDs will not appear in the published article. *Only*  the individual author can complete the verification step; PLOS staff *cannot*  verify ORCID iDs on behalf of authors.

We look forward to receiving your revised manuscript.

Kind regards,

Jennifer Tucker, PhD

Staff Editor

PLOS One

**Journal Requirements:**

When submitting your revision, we need you to address these additional requirements.

1. Please ensure that your manuscript meets PLOS ONE's style requirements, including those for file naming. The PLOS ONE style templates can be found at

https://journals.plos.org/plosone/s/file?id=wjVg/PLOSOne_formatting_sample_main_body.pdf and

https://journals.plos.org/plosone/s/file?id=ba62/PLOSOne_formatting_sample_title_authors_affiliations.pdf

2. Please include your full ethics statement in the ‘Methods’ section of your manuscript file. In your statement, please include the full name of the IRB or ethics committee who approved or waived your study, as well as whether or not you obtained informed written or verbal consent. If consent was waived for your study, please include this information in your statement as well.

3. Please upload a new copy of Figures 1, 2 and 3 as the detail is not clear. Please follow the link for more information:  https://journals.plos.org/plosone/s/figures

4. Please include captions for your Supporting Information files at the end of your manuscript, and update any in-text citations to match accordingly. Please see our Supporting Information guidelines for more information: http://journals.plos.org/plosone/s/supporting-information.

5. If the reviewer comments include a recommendation to cite specific previously published works, please review and evaluate these publications to determine whether they are relevant and should be cited. There is no requirement to cite these works unless the editor has indicated otherwise.

[Note: HTML markup is below. Please do not edit.]

Reviewers' comments:

Reviewer's Responses to Questions

**Comments to the Author**

1. Is the manuscript technically sound, and do the data support the conclusions?

Reviewer #1: Yes

Reviewer #2: Yes

Reviewer #3: Yes

**********

2. Has the statistical analysis been performed appropriately and rigorously?

Reviewer #1: Yes

Reviewer #2: Yes

Reviewer #3: Yes

**********

3. Have the authors made all data underlying the findings in their manuscript fully available?

The PLOS Data policy

Reviewer #1: Yes

Reviewer #2: Yes

Reviewer #3: Yes

**********

4. Is the manuscript presented in an intelligible fashion and written in standard English?

Reviewer #1: Yes

Reviewer #2: Yes

Reviewer #3: Yes

**********

Reviewer #1: This manuscript addresses an important and timely topic: pediatric emergency medicine (PEM) preparedness in Lebanon, a setting where structured PEM training pathways remain underdeveloped. The study provides valuable insights into physicians’ self-reported comfort levels, perceived challenges, and training needs, and offers a meaningful contribution to the literature on emergency care in resource-constrained settings.

Overall, the study design and analytical approach are appropriate for the research objectives. The use of a cross-sectional survey and comparison of proportions between general pediatricians and emergency-focused physicians using Chi-square or Fisher’s exact tests is suitable for the type of data collected. The findings are generally clearly presented and clinically coherent. In particular, the observed differences—where general pediatricians report higher confidence in general pediatric emergencies, and emergency-focused physicians demonstrate higher confidence in trauma-related and procedural skills—are consistent with expected differences in training and clinical exposure, supporting the internal validity of the results.

That said, a few minor clarifications and improvements would strengthen the manuscript:

1- Clarification of Data Handling:

A discrepancy exists between the Data Analysis (Section 2.5) and the results presented in Tables 2 and 3. The Methods section states that ordinal variables (such as Likert-scale data) would be presented as means and standard deviations. However, the tables instead report the proportion of respondents who were “comfortable” or “extremely comfortable.”To ensure internal consistency, the Methods section should be updated to explicitly state that the Likert-scale responses were dichotomized into binary categories for analysis and presentation.

2- Statistical Reporting Transparency:

While the manuscript indicates that Chi-square or Fisher’s exact tests were used “as appropriate,” specifying which test was applied in each comparison (particularly where cell counts are small) would enhance transparency. It is best practice to use a footnote symbol (like * or †) on specific p-values in the tables to show exactly which test was used for each specific row.

3- Minor Typographical and Formatting Issues:

There are a few small errors in Table 2 (e.g., “0..007” and formatting inconsistencies) that should be corrected for clarity and professionalism.

4- Clarity and Conciseness:

The Results section is generally well written, though minor reductions in repetition (especially where numerical values are already presented in tables) could improve readability.

Overall, this is a well-conducted and relevant study that provides useful descriptive and comparative insights into pediatric emergency care capacity in Lebanon. With minor revisions to improve clarity and presentation, the manuscript would be suitable for publication.

Reviewer #2: This is an interesting work allowing to better understand the views of clinicians working in paediatric emergency departments in Lebanon.

However I have majors comments :

- We do not know the denominator corresponding to the number of physicians working in paediatric emergency departments in Lebanon. Without this information, or an approximate one at least, it is impossible to draw any conclusions on the validity of the results. This number should be given to the reader.

- It is surprising that the authors state that there is no PEM specialist registered in Lebanon although there is 8 in their respondents.

- Regarding the discussion, if there is such important lacks of competence among physicians actually working in emergency departments (few POCUS training, difficulties to read X-rays etc), how does the authors imagine the creation of a PEM fellowship in Lebanon? Different teaching pathways may be needed: continuing medical education at first to increase the level of care and confidence of physicians already working in the PEDs, fellowship to train the younger clinicians.

It would also be interesting to know more about the work division between PEM and PICU physicians in Lebanon.

Reviewer #3: Summary

This manuscript presents a cross-sectional survey evaluating pediatric emergency medicine (PEM) preparedness, physician comfort, and training needs in Lebanon. The study addresses an important and underexplored topic, particularly in a context where structured PEM systems remain limited. The findings highlight perceived gaps in training and confidence among physicians and suggest the need for further educational and system-level development.

More broadly, the study aligns with existing literature demonstrating that emergency care systems in low- and middle-income countries (LMICs) often face similar challenges, including limited subspecialty training, reliance on generalists, and variability in preparedness (Rybarczyk et al., 2020). While the manuscript provides useful preliminary insights, several methodological and interpretative issues should be addressed to strengthen its contribution.

Major Comments

1. Global Context in the Introduction

The introduction appropriately describes the development of PEM in North America and Europe. However, it would benefit from a more comprehensive global perspective.

Specifically, the authors are encouraged to include the expansion of PEM training programs in Latin America and Australia, where structured educational initiatives and fellowships have developed over the past decade. Including these regions would provide a more balanced global overview and better situate Lebanon within the broader international evolution of PEM.

2. Sampling Strategy and Representativeness

The study uses convenience sampling, and the total number of physicians invited to participate is not reported. As a result:

• The response rate cannot be determined

• The sampling frame is unclear

• Representativeness cannot be assessed

The statement that the sample represents “a substantial portion of the reachable pediatric emergency workforce in Lebanon” is not sufficiently supported by the data presented.

The authors should:

• Provide estimates of the national workforce (if available)

• Either justify or rephrase claims of representativeness

3. Reliance on Self-Reported Measures

The study relies on self-reported comfort levels, which may not accurately reflect clinical competence.

• Prior research shows discrepancies between confidence and actual performance (Hoysted et al., 2018)

• Self-report introduces potential reporting and recall bias

The authors should:

• Emphasize this limitation more explicitly

• Avoid interpreting “comfort” as equivalent to preparedness or competence

4. Lack of Outcome-Based Measures

The manuscript does not include objective clinical outcomes or performance indicators.

• This limits the ability to link perceived preparedness to quality of care or patient outcomes

• Similar studies emphasize the importance of outcome-based validation (Rybarczyk et al., 2020)

Recommendation:

Reframe conclusions to reflect that this is a perception-based needs assessment, not an evaluation of care quality.

Strengths

Despite its limitations, this study has several important strengths:

• It addresses a significant gap in the literature, particularly in Lebanon

• Provides rare, context-specific data on PEM workforce perceptions

• Includes physicians from multiple relevant specialties

• Functions as a needs assessment, which is a recognized first step in health system development

Importantly, in settings with limited existing data, exploratory studies like this can play a meaningful role in:

• Identifying system gaps

• Informing future research

• Supporting academic and institutional dialogue

Such contributions are widely recognized as essential for the development of emergency care systems in LMICs (Reynolds et al., 2013; Razzak et al., 2019).

Suggested Revision – Strengths and Limitations Section

The authors may consider revising this section as follows:

This study addresses an important gap in the literature by providing one of the first assessments of pediatric emergency medicine (PEM) preparedness and training needs among physicians in Lebanon. In a context where published data are limited, such exploratory, needs-based assessments offer valuable preliminary insights into workforce perceptions, potential skill gaps, and priority areas for educational development. The inclusion of physicians from multiple specialties involved in pediatric emergency care further broadens the perspective.

However, several limitations should be acknowledged. The use of convenience sampling and the absence of a clearly defined sampling frame limit the ability to assess representativeness. The total number of physicians invited to participate is not reported, and therefore the response rate cannot be determined, introducing potential non-response bias.

In addition, the reliance on self-reported measures of comfort and preparedness may not accurately reflect actual clinical competence. The relatively small sample size (n = 59) further limits statistical power and generalizability.

These findings should therefore be interpreted as exploratory and hypothesis-generating. Future studies using larger, systematically sampled populations and objective assessments would strengthen the evidence base.

**********

what does this mean?). If published, this will include your full peer review and any attached files.

If you choose “no”, your identity will remain anonymous but your review may still be made public.

**Do you want your identity to be public for this peer review?**  For information about this choice, including consent withdrawal, please see our Privacy Policy

Reviewer #1: No

Reviewer #2: No

Reviewer #3: No

**********

[NOTE: If reviewer comments were submitted as an attachment file, they will be attached to this email and accessible via the submission site. Please log into your account, locate the manuscript record, and check for the action link "View Attachments". If this link does not appear, there are no attachment files.]

To ensure your figures meet our technical requirements, please review our figure guidelines: https://journals.plos.org/plosone/s/figures

You may also use PLOS’s free figure tool, NAAS, to help you prepare publication quality figures: https://journals.plos.org/plosone/s/figures#loc-tools-for-figure-preparation.

NAAS will assess whether your figures meet our technical requirements by comparing each figure against our figure specifications.

# Author comment: Author response to Decision Letter 1

**DOI:** 10.1371/journal.pone.0361212.r002

18 May 2026

Reviewer #1:

This manuscript addresses an important and timely topic: pediatric emergency medicine (PEM) preparedness in Lebanon, a setting where structured PEM training pathways remain underdeveloped. The study provides valuable insights into physicians’ self-reported comfort levels, perceived challenges, and training needs, and offers a meaningful contribution to the literature on emergency care in resource-constrained settings.

Overall, the study design and analytical approach are appropriate for the research objectives. The use of a cross-sectional survey and comparison of proportions between general pediatricians and emergency-focused physicians using Chi-square or Fisher’s exact tests is suitable for the type of data collected. The findings are generally clearly presented and clinically coherent. In particular, the observed differences—where general pediatricians report higher confidence in general pediatric emergencies, and emergency-focused physicians demonstrate higher confidence in trauma-related and procedural skills—are consistent with expected differences in training and clinical exposure, supporting the internal validity of the results. That said, a few minor clarifications and improvements would strengthen the manuscript:

1- Clarification of Data Handling:

A discrepancy exists between the Data Analysis (Section 2.5) and the results presented in Tables 2 and 3. The Methods section states that ordinal variables (such as Likert-scale data) would be presented as means and standard deviations. However, the tables instead report the proportion of respondents who were “comfortable” or “extremely comfortable.” To ensure internal consistency, the Methods section should be updated to explicitly state that the Likert-scale responses were dichotomized into binary categories for analysis and presentation.

Author’s Reply: Thank you for your valuable comment. To clarify, Likert-scale responses were presented descriptively in the tables as proportions across all response categories. For the purpose of inferential analysis, these variables were recoded into binary categories (e.g., “comfortable/extremely comfortable” versus other responses) to facilitate statistical testing. The Data Analysis section has been revised accordingly to clearly describe this approach and ensure consistency between the Methods and the results.

2- Statistical Reporting Transparency: While the manuscript indicates that Chi-square or Fisher’s exact tests were used “as appropriate,” specifying which test was applied in each comparison (particularly where cell counts are small) would enhance transparency. It is best practice to use a footnote symbol (like * or †) on specific p-values in the tables to show exactly which test was used for each specific row.

Author’s Reply: Thank you for this helpful suggestion. We agree that specifying the statistical test used for each comparison improves transparency. Accordingly, the tables have been revised to indicate the test applied for each p-value using appropriate footnote symbols (e.g., * for Chi-square test and † for Fisher’s exact test).

3- Minor Typographical and Formatting Issues:

There are a few small errors in Table 2 (e.g., “0.007” and formatting inconsistencies) that should be corrected for clarity and professionalism.

Author’s Reply: Corrected

4- Clarity and Conciseness:

The Results section is generally well written, though minor reductions in repetition (especially where numerical values are already presented in tables) could improve readability.

Author’s Reply: We have reviewed the Results section and reduced minor repetition by removing redundant textual descriptions of numerical values already presented in the tables.

Overall, this is a well-conducted and relevant study that provides useful descriptive and comparative insights into pediatric emergency care capacity in Lebanon. With minor revisions to improve clarity and presentation, the manuscript would be suitable for publication.

Author’s Reply: We sincerely thank the reviewer for the positive assessment of our work and for recognizing its relevance to pediatric emergency care capacity in Lebanon. We appreciate the constructive feedback provided throughout the review process, which has helped us improve the clarity and presentation of the manuscript. We have carefully addressed all comments and revised the manuscript accordingly.

Reviewer #2:

This is an interesting work allowing to better understand the views of clinicians working in paediatric emergency departments in Lebanon. However I have majors comments:

- We do not know the denominator corresponding to the number of physicians working in paediatric emergency departments in Lebanon. Without this information, or an approximate one at least, it is impossible to draw any conclusions on the validity of the results. This number should be given to the reader.

Author’s Reply: We thank the reviewer for this important comment. We agree that providing the total number of physicians working in pediatric emergency care in Lebanon would help contextualize the representativeness of our sample. However, no official or publicly available registry exists that precisely defines the number of physicians working in pediatric emergency departments nationwide, and staffing patterns vary across institutions and specialties. To address this limitation, we have clarified in the manuscript that the total national denominator is not available and have explicitly stated this as a limitation of the study. We have also expanded the discussion to emphasize that the findings should be interpreted as reflecting the perspectives of the participating physicians rather than a nationally representative sample.

- It is surprising that the authors state that there is no PEM specialist registered in Lebanon although there is 8 in their respondents.

Author’s Reply: The apparent discrepancy arises from differences in terminology and professional classification. In Lebanon, there is currently no formally accredited national subspecialty certification or official registry for PEM as a recognized specialty.

The physicians referred to as PEM in our study are clinicians who self-identified as having focused training or clinical practice in pediatric emergency or pediatric critical care settings, rather than formally registered PEM specialists in a nationally recognized subspecialty program. This has now been clarified in the manuscript to avoid ambiguity.

- Regarding the discussion, if there is such important lacks competence among physicians actually working in emergency departments (few POCUS training, difficulties to read X-rays etc), how does the authors imagine the creation of a PEM fellowship in Lebanon? Different teaching pathways may be needed: continuing medical education at first to increase the level of care and confidence of physicians already working in the PEDs, fellowship to train the younger clinicians.

Author’s Reply: We fully agree that the establishment of a PEM fellowship should be part of a broader, stepwise strategy rather than a stand-alone intervention. As highlighted by our findings, the presence of important gaps in procedural and diagnostic competencies among physicians currently working in pediatric emergency departments supports the need for immediate capacity-building measures. Accordingly, we have revised the Discussion to emphasize a dual approach: short-term continuing medical education (CME) programs aimed at improving current clinical competencies (e.g., POCUS, radiograph interpretation, and procedural skills), alongside the long-term development of a structured PEM fellowship to train future specialists. This integrated approach is now clearly reflected in the revised manuscript.

It would also be interesting to know more about the work division between PEM and PICU physicians in Lebanon.

Author’s Reply: We have addressed this point in the Discussion by highlighting the complementary roles of PEM physicians in the initial assessment, resuscitation, and stabilization of critically ill children, and PICU physicians in providing ongoing intensive care after admission. We also incorporated a recommendation emphasizing the need for clear role delineation, shared clinical protocols, and joint training initiatives to strengthen coordination and ensure continuity of care between these two specialties in Lebanon.

Reviewer #3:

Summary

This manuscript presents a cross-sectional survey evaluating pediatric emergency medicine (PEM) preparedness, physician comfort, and training needs in Lebanon. The study addresses an important and underexplored topic, particularly in a context where structured PEM systems remain limited. The findings highlight perceived gaps in training and confidence among physicians and suggest the need for further educational and system-level development. More broadly, the study aligns with existing literature demonstrating that emergency care systems in low- and middle-income countries (LMICs) often face similar challenges, including limited subspecialty training, reliance on generalists, and variability in preparedness (Rybarczyk et al., 2020). While the manuscript provides useful preliminary insights, several methodological and interpretative issues should be addressed to strengthen its contribution.

Major Comments

1. Global Context in the Introduction

The introduction appropriately describes the development of PEM in North America and Europe. However, it would benefit from a more comprehensive global perspective. Specifically, the authors are encouraged to include the expansion of PEM training programs in Latin America and Australia, where structured educational initiatives and fellowships have developed over the past decade. Including these regions would provide a more balanced global overview and better situate Lebanon within the broader international evolution of PEM.

Author’s Reply: Thank you for this insightful suggestion. We have revised the introduction to include the development of Pediatric Emergency Medicine (PEM) in Latin America and Australia/New Zealand. Specifically, we added information regarding the emergence of structured PEM training programs, fellowships, and collaborative research initiatives such as the Pediatric Research in Emergency Departments International Collaborative (PREDICT), as well as the expansion and recognition of PEM programs across several Latin American countries. These additions provide a more comprehensive global perspective on the evolution of PEM.

2. Sampling Strategy and Representativeness

The study uses convenience sampling, and the total number of physicians invited to participate is not reported. As a result:

• The response rate cannot be determined

• The sampling frame is unclear

• Representativeness cannot be assessed

The statement that the sample represents “a substantial portion of the reachable pediatric emergency workforce in Lebanon” is not sufficiently supported by the data presented.

The authors should:

• Provide estimates of the national workforce (if available)

• Either justify or rephrase claims of representativeness

Author’s Reply: We have revised the manuscript to remove the statement suggesting that the sample represents a “substantial portion” of the pediatric emergency workforce. We have clarified in the Methods section that the total number of physicians invited is unknown and that no official registry exists to define the national pediatric emergency workforce. These limitations are now explicitly acknowledged, and the findings are presented reflecting the perspectives of participating physicians rather than as nationally representative estimates.

3. Reliance on Self-Reported Measures

The study relies on self-reported comfort levels, which may not accurately reflect clinical competence.

• Prior research shows discrepancies between confidence and actual performance (Hoysted et al., 2018)

• Self-report introduces potential reporting and recall bias

The authors should:

• Emphasize this limitation more explicitly

• Avoid interpreting “comfort” as equivalent to preparedness or competence

Author’s Reply: Thank you for this important comment. We agree that self-reported comfort does not necessarily reflect actual clinical competence. Accordingly, we have revised the manuscript throughout to clarify that the study assesses perceived comfort only, and we have avoided interpreting comfort as equivalent to preparedness or competence. We have also strengthened the limitations section to explicitly acknowledge the potential for reporting and recall bias associated with self-reported measures.

4. Lack of Outcome-Based Measures The manuscript does not include objective clinical outcomes or performance indicators.

• This limits the ability to link perceived preparedness to quality of care or patient outcomes

• Similar studies emphasize the importance of outcome-based validation (Rybarczyk et al., 2020)

Recommendation:

Reframe conclusions to reflect that this is a perception-based needs assessment, not an evaluation of care quality.

Author’s Reply: We have revised the Conclusion to clearly reflect that this study represents a perception-based needs assessment rather than an evaluation of clinical performance or care quality. We have also made statements that could imply outcome-level impact and emphasized that findings are based on reported comfort levels. In addition, we have highlighted the need for future research incorporating objective outcome measures and performance-based assessments.

Strengths

Despite its limitations, this study has several important strengths:

• It addresses a significant gap in the literature, particularly in Lebanon

• Provides rare, context-specific data on PEM workforce perceptions

• Includes physicians from multiple relevant specialties

• Functions as a needs assessment, which is a recognized first step in health system development

Importantly, in settings with limited existing data, exploratory studies like this can play a meaningful role in:

• Identifying system gaps

• Informing future research

• Supporting academic and institutional dialogue

Such contributions are widely recognized as essential for the development of emergency care systems in LMICs (Reynolds et al., 2013; Razzak et al., 2019).

Suggested Revision – Strengths and Limitations Section

The authors may consider revising this section as follows:

This study addresses an important gap in the literature by providing one of the first assessments of pediatric emergency medicine (PEM) preparedness and training needs among physicians in Lebanon. In a context where published data are limited, such exploratory, needs-based assessments offer valuable preliminary insights into workforce perceptions, potential skill gaps, and priority areas for educational development. The inclusion of physicians from multiple specialties involved in pediatric emergency care further broadens the perspective.

However, several limitations should be acknowledged. The use of convenience sampling and the absence of a clearly defined sampling frame limit the ability to assess representativeness. The total number of physicians invited to participate is not reported, and therefore the response rate cannot be determined, introducing potential non-response bias.

In addition, the reliance on self-reported measures of comfort and preparedness may not accurately reflect actual clinical competence. The relatively small sample size (n = 59) further limits statistical power and generalizability.

These findings should therefore be interpreted as exploratory and hypothesis-generating. Future studies using larger, systematically sampled populations and objective assessments would strengthen the evidence base.

Author’s Reply: We thank the reviewer for this thoughtful and constructive suggestion. We appreciate the revised framing of the Strengths and Limitations section, which improves clarity and better reflects the exploratory nature of the study. We have revised the manuscript accordingly, incorporating the suggested structure and wording with minor adaptations to maintain consistency with the overall manuscript style.

Attachment Submitted filename: Response to reviewer.docx

# Peer review: Decision Letter 1

**DOI:** 10.1371/journal.pone.0361212.r003

**Authors:** Gayle E. Woloschak

5 Oct 2026

Dear Dr. Moughnieh,

Thank you for submitting your manuscript to PLOS One. After careful consideration, we feel that it has merit but does not fully meet PLOS One’s publication criteria as it currently stands. Therefore, we invite you to submit a revised version of the manuscript that addresses the points raised during the review process.

Please submit your revised manuscript by Nov 19 2026 11:59PM. If you will need more time than this to complete your revisions, please reply to this message or contact the journal office at plosone@plos.org. When you're ready to submit your revision, log on to https://www.editorialmanager.com/pone/ and select the 'Submissions Needing Revision' folder to locate your manuscript file.

- A letter that responds to each point raised by the academic editor and reviewer(s). You should upload this letter as a separate file labeled 'Response to Reviewers'.
- A marked-up copy of your manuscript that highlights changes made to the original version. You should upload this as a separate file labeled 'Revised Manuscript with Track Changes'.
- An unmarked version of your revised paper without tracked changes. You should upload this as a separate file labeled 'Manuscript'.

If you would like to make changes to your financial disclosure, please include your updated statement in your cover letter. Guidelines for resubmitting your figure files are available below the reviewer comments at the end of this letter.

If applicable, we recommend that you deposit your laboratory protocols in protocols.io to enhance the reproducibility of your results. Protocols.io assigns your protocol its own identifier (DOI) so that it can be cited independently in the future. For instructions see: https://journals.plos.org/plosone/s/submission-guidelines#loc-laboratory-protocols. Additionally, PLOS One offers an option for publishing peer-reviewed Lab Protocol articles, which describe protocols hosted on protocols.io. Read more information on sharing protocols at https://plos.org/protocols?utm_medium=editorial-email&utm_source=authorletters&utm_campaign=protocols.

As the corresponding author, your ORCID iD is verified in the submission system and will appear in the published article. PLOS supports the use of ORCID, and we encourage all coauthors to register for an ORCID iD and use it as well. Please encourage your coauthors to verify their ORCID iD within the submission system before final acceptance, as unverified ORCID iDs will not appear in the published article. *Only*  the individual author can complete the verification step; PLOS staff *cannot*  verify ORCID iDs on behalf of authors.

We look forward to receiving your revised manuscript.

Kind regards,

Gayle E. Woloschak, PhD

Section Editor

PLOS One

Journal Requirements:

If the reviewer comments include a recommendation to cite specific previously published works, please review and evaluate these publications to determine whether they are relevant and should be cited. There is no requirement to cite these works unless the editor has indicated otherwise.

Please review your reference list to ensure that it is complete and correct. If you have cited papers that have been retracted, please include the rationale for doing so in the manuscript text, or remove these references and replace them with relevant current references. Any changes to the reference list should be mentioned in the rebuttal letter that accompanies your revised manuscript. If you need to cite a retracted article, indicate the article’s retracted status in the References list and also include a citation and full reference for the retraction notice.

Additional Editor Comments:

One reviewer has suggested additional minor revisions. Obviously the survey cannot be changed at this point, but perhaps addressing the issues raised in the text can improve the work.

[Note: HTML markup is below. Please do not edit.]

Reviewers' comments:

Reviewer's Responses to Questions

**Comments to the Author**

Reviewer #2: All comments have been addressed

Reviewer #4: All comments have been addressed

**********

2. Is the manuscript technically sound, and do the data support the conclusions?

Reviewer #2: Yes

Reviewer #4: Partly

**********

3. Has the statistical analysis been performed appropriately and rigorously?

Reviewer #2: Yes

Reviewer #4: Yes

**********

4. Have the authors made all data underlying the findings in their manuscript fully available?

The PLOS Data policy

Reviewer #2: Yes

Reviewer #4: Yes

**********

5. Is the manuscript presented in an intelligible fashion and written in standard English?

Reviewer #2: Yes

Reviewer #4: Yes

**********

Reviewer #2: the authors have carefully revised the manuscript according to reviewers comments. They improved the validity and understanding of their findings.

Reviewer #4: I have two comments; one reiterates previous ones and one in addition, and two questions:

1. Regarding the number of sampled physicians, why the number of recipients in whatsapp, or the total number of members in medical societies tallied? That would at least give an approximate number of survey recipients? As it stands, the small number of responders may be interpreted as selection bias by the authors or participants themselves.

2. Considering the small number of total respondents, the differences highlighted in tables 2 and 3 are difficult to interpret. I suggest treating this work as a descriptive study and eliminate the analyses.

3. How was PEM defined for the potential survey responders in the dissemination material? That may have led to some providers self-selecting to complete the survey if they felt they did not fit that definition, particularly in a country with no defined certification in PEM.

4. Why didn't the authors ask in the survey how many years in total practice the providers have had, as that could be a correlation for how they came about working in PEM, especially if there is no dedicated training program?

Even as a descriptive study, this work highlights an area of need in a region of the world that struggles in the delivery of healthcare services.

**********

what does this mean?). If published, this will include your full peer review and any attached files.

If you choose “no”, your identity will remain anonymous but your review may still be made public.

**Do you want your identity to be public for this peer review?**  For information about this choice, including consent withdrawal, please see our Privacy Policy

Reviewer #2: No

Reviewer #4: No

**********

[NOTE: If reviewer comments were submitted as an attachment file, they will be attached to this email and accessible via the submission site. Please log into your account, locate the manuscript record, and check for the action link "View Attachments". If this link does not appear, there are no attachment files.]

To ensure your figures meet our technical requirements, please review our figure guidelines: https://journals.plos.org/plosone/s/figures

You may also use PLOS’s free figure tool, NAAS, to help you prepare publication quality figures: https://journals.plos.org/plosone/s/figures#loc-tools-for-figure-preparation.

NAAS will assess whether your figures meet our technical requirements by comparing each figure against our figure specifications.

# Author comment: Author response to Decision Letter 2

**DOI:** 10.1371/journal.pone.0361212.r004

6 Oct 2026

Dear Editor,

We sincerely thank you and Reviewer #4 for the thoughtful and constructive comments on our manuscript. The reviewer’s comments were particularly helpful in strengthening the methodological transparency of the study and ensuring that the interpretation of the findings is appropriately aligned with the study design and sample size.

In response to the comments, we have made the following major revisions to the manuscript:

Sampling and response denominator: We clarified that, because the survey was disseminated through multiple overlapping channels, the number of unique physicians who received the invitation could not be determined. We therefore did not calculate a response rate and have strengthened the discussion of the limitations related to sampling representativeness and potential selection bias.

Descriptive analysis: In accordance with the reviewer’s recommendation, we have revised the study to be presented as a descriptive cross-sectional survey. The inferential analyses and associated p-values in Tables 2 and 3 have been removed. These tables now present the findings descriptively using frequencies and percentages. The Methods, Results, and Discussion have also been revised accordingly to ensure that no statistical significance is implied by the observed subgroup differences.

Definition of PEM and participant self-selection: We clarified the eligibility criteria and recruitment approach in the Methods. Given the absence of a nationally recognized PEM certification or registry in Lebanon, participation relied on physicians’ self-identification as being involved in pediatric emergency care. We have explicitly acknowledged the potential for self-selection as a limitation and have avoided interpreting the sample as representative of all physicians providing pediatric emergency care in Lebanon.

Years of clinical experience: We clarified that years of clinical experience were collected in the survey and are reported in Table 1. The median duration of clinical experience among the 59 respondents was 7 years (IQR: 5; range: 1–30 years). Given the revised descriptive approach and limited sample size, we did not perform additional correlation or inferential analyses.

We also appreciate the reviewer’s positive observation that, even as a descriptive study, the work highlights an important area of need in pediatric emergency care. We have strengthened the manuscript to emphasize the educational and health-system needs identified by the participating physicians while maintaining a cautious interpretation and avoiding generalization beyond the study sample.

We are grateful for the opportunity to revise the manuscript and for the valuable guidance provided by the reviewer and the editorial team. We believe that the revisions have improved the methodological clarity, transparency, and overall interpretation of the manuscript, and we hope that the revised version will now be suitable for publication.

Thank you again for your time and consideration of our work.

Sincerely,

Nabil Moghnieh

On behalf of all authors

Attachment Submitted filename: Response to Reviewers.docx

# Editor report: Decision Letter 2

**DOI:** 10.1371/journal.pone.0361212.r005

**Authors:** Gayle E. Woloschak

8 Oct 2026

Pediatric Emergency Medicine in Lebanon: Physician-Perceived Comfort Levels, Challenges, and Educational Needs

PONE-D-26-08269R2

Dear Dr. Moughnieh,

We’re pleased to inform you that your manuscript has been judged scientifically suitable for publication and will be formally accepted for publication once it meets all outstanding technical requirements.

Within one week, you’ll receive an e-mail detailing the required amendments. When these have been addressed, you’ll receive a formal acceptance letter and your manuscript will be scheduled for publication.

An invoice will be generated when your article is formally accepted. Please note, if your institution has a publishing partnership with PLOS and your article meets the relevant criteria, all or part of your publication costs will be covered. Please make sure your user information is up-to-date by logging into Editorial Manager at Editorial Manager® and clicking the ‘Update My Information' link at the top of the page. For questions related to billing, please contact billing support.

If your institution or institutions have a press office, please notify them about your upcoming paper to help maximize its impact. If they’ll be preparing press materials, please inform our press team as soon as possible -- no later than 48 hours after receiving the formal acceptance. Your manuscript will remain under strict press embargo until 2 pm Eastern Time on the date of publication. For more information, please contact onepress@plos.org.

Kind regards,

Gayle E. Woloschak, PhD

Section Editor

PLOS One

Additional Editor Comments (optional):

Thank you for addressing the concerns of the reviewer.

Reviewers' comments:

# Editor report: Acceptance letter

**DOI:** 10.1371/journal.pone.0361212.r006

**Authors:** Gayle E. Woloschak

PONE-D-26-08269R2

PLOS One

Dear Dr. Moughnieh,

I'm pleased to inform you that your manuscript has been deemed suitable for publication in PLOS One. Congratulations! Your manuscript is now being handed over to our production team.

At this stage, our production department will prepare your paper for publication. This includes ensuring the following:

* All references, tables, and figures are properly cited

* All relevant supporting information is included in the manuscript submission,

* There are no issues that prevent the paper from being properly typeset

You will receive further instructions from the production team, including instructions on how to review your proof when it is ready. Please keep in mind that we are working through a large volume of accepted articles, so please give us a few days to review your paper and let you know the next and final steps.

Lastly, if your institution or institutions have a press office, please let them know about your upcoming paper now to help maximize its impact. If they'll be preparing press materials, please inform our press team within the next 48 hours. Your manuscript will remain under strict press embargo until 2 pm Eastern Time on the date of publication. For more information, please contact onepress@plos.org.

You will receive an invoice from PLOS for your publication fee after your manuscript has reached the completed accept phase. If you receive an email requesting payment before acceptance or for any other service, this may be a phishing scheme. Learn how to identify phishing emails and protect your accounts at https://explore.plos.org/phishing.

If we can help with anything else, please email us at customercare@plos.org.

Thank you for submitting your work to PLOS One and supporting open access.

Kind regards,

PLOS One Editorial Office Staff

on behalf of

Dr. Gayle E. Woloschak

Section Editor

PLOS One
